# Analysis of Non‐Coding RNAs and N6‐Methyladenosine‐Modified Genes in Response to flg22 in Grape Immunity

**DOI:** 10.1111/pbi.70303

**Published:** 2025-08-17

**Authors:** Ruiwei Duan, Yang Feng, Runxin Zhang, Wenjing Lu, Qingqing Xie, Dong Duan

**Affiliations:** ^1^ Key Laboratory of Resource Biology and Biotechnology in Western China, Ministry of Education, College of Life Sciences Northwest University Xi'an China

**Keywords:** disease resistance, m^6^A, plant‐pathogen interaction, *V*. *quinquangularis*, whole transcriptome‐seq

## Abstract

Grape ( *Vitis vinifera* ) is susceptible to fungal pathogens and, consequently, severe yield losses. Investigating the immune mechanisms of the disease‐resistant Chinese wild grapes is crucial for developing sustainable disease resistance technologies. Here, we conducted whole‐transcriptome and methylated‐RNA‐immunoprecipitation sequencing to elucidate the immune defence mechanisms underlying grapevine responses to bacterial flagellin 22 (flg22). Certain differentially expressed miRNAs and lncRNAs responsive to flg22 showed greater differences in genotype *‘*Shanyang’ than those in ‘Cabernet Sauvignon’. Functional annotation of miRNA target genes revealed that ‘Shanyang’‐specific pathways were associated with ethylene‐activated signalling, etc. Additionally, the *cis*‐target genes of lncRNAs were significantly enriched in the trihydroxystilbene synthase activity, etc. Notably, transient overexpression of lncRNAs TCONS_00015412, TCONS_00070812, and TCONS_00070833 in *V. quinquangularis* conferred significantly enhanced disease resistance compared to control plants. Significantly different m^6^A peaks were located in the coding sequence and stop‐codon regions. Integrated analysis of m^6^A and RNA‐seq suggested that m^6^A methylation within the coding sequence generally enhanced mRNA expression. Functional analysis further demonstrated that significantly differentially expressed genes with differential m^6^A modifications were enriched in the plant‐pathogen interaction pathway, etc. Furthermore, among m^6^A‐modified genes, *LRR‐RLK* in *V. quinquangularis* was confirmed to enhance grapevine resistance to *C. diplodiella* through transient overexpression. Altogether, our data strongly indicate that m^6^A methylation and non‐coding RNAs regulated immune‐related gene expression upon flg22 treatment, thereby modulating grapevine defence signalling pathways against pathogens. These results significantly enhance our understanding of the molecular mechanisms involving non‐coding RNAs and m^6^A genes in the grapevine immune‐defence responses and provide a valuable theoretical foundation for grapevine resistance breeding.

Plants have evolved a two‐tiered immune system through long‐term host‐pathogen interactions, primarily consisting of pattern‐triggered immunity (PTI) and effector‐triggered immunity (ETI) (Jones and Dangl 2006), to counteract pathogen invasion and persistent threats (Macho and Zipfel 2014). Within the PTI system, plants have developed various types of receptor‐like kinases to recognise pathogens, among which leucine‐rich repeat sequence receptor kinases (*LRR‐RLK*s) constitute the largest family (Boller and Felix 2009). A well‐characterised example is the *Arabidopsis* leucine‐rich repeat receptor kinases FLAGELLIN SENSING 2 (*FLS2*), which specifically recognises the bacterial flagellin (flg22 as derived peptide). Upon binding to flg22, *FLS2* rapidly forms a complex with the co‐receptor brassinosteroid insensitive 1‐associated receptor kinase 1 (*BAK1*), initiating downstream immune responses (Denoux et al. 2008). These responses include the production of reactive oxygen species (ROS), Ca^2+^ influx, and mitogen‐activated protein kinase (MAPK) cascade. Additionally, the immune response encompasses the accumulation of secondary metabolites, alterations in cell wall composition, and plant hormone signal transduction pathways (Bigeard et al. 2015).

Grapes are widely cultivated worldwide for their high quality, yet most commercial varieties ( *Vitis vinifera* ) remain susceptible to devastating fungal pathogens including powdery mildew (Calonnec et al. 2021), white rot (Li et al. 2023) and downy mildew, which compromise berry quality and leaf photosynthetic capacity (Duan et al. 2015). China, as one of the original regions of wild grapes, harbours abundant disease‐resistant resources, such as Chinese wild grape *V. quinquangularis* ‘Shanyang’ (Jiang et al. 2023), *V. quinquangularis* ‘Danfeng‐2’ (Liu et al. 2023) and *V. pseudoreticulata* ‘Baihe 35‐1’ (Liu et al. 2015), and provides valuable disease‐resistant germplasm for addressing limited resistance genes in cultivated grapes. While researchers have identified multiple resistance loci to combat pathogen invasion, such as powdery mildew resistance loci *Ren1*, *Ren3*, and *Ren9* (Massonnet et al. 2022; Zendler et al. 2021), and *Rpv3* and *Rpv1* against downy mildew (Bellin et al. 2009; Peressotti et al. 2010). However, these often prove unstable—as demonstrated when *avrRpv3* mutants overcame *Rpv3*‐mediated resistance (Peressotti et al. 2010). Notably, pattern‐triggered immunity (PTI) emerges as a robust defence mechanism, evidenced by European wild grape ( *V. vinifera ssp. sylvestris* ) resistance to downy mildew (Duan et al. 2016) and Chinese wild grape defences involving stilbene synthesis (*VpSTS*/*VqMYB15*) that enhance SA signalling and pathogen resistance (Xu et al. 2010; Luo et al. 2019). These findings underscore the importance of investigating plant immunity in wild grape resources to develop durable resistance strategies against evolving pathogens (Bigeard et al. 2015).

Recent progress in high‐throughput sequencing has enabled extensive use of whole‐transcriptome sequencing to elucidate biological processes and disease resistance mechanisms in plants, such as *Arabidopsis* (Zhu et al. 2014), wheat (Shumayla et al. 2017), tomato (Wang et al. 2015), and *V. quinquangularis* (Jiang et al. 2023), among other plant species. Non‐coding RNAs (ncRNAs)—including miRNAs, lncRNAs, and circRNAs—orchestrate stress responses by dynamically regulating gene expression (Hou et al. 2019). LncRNAs are transcripts (> 200 nt) with no protein‐coding capacity, and they exhibit features distinct from mRNAs, such as lower expression levels, weaker sequence conservation, and tissue‐specific expression patterns (Liu, Wang, and Chua 2015; Shafiq et al. 2016). LncRNAs can localise in the nucleus or cytoplasm and execute their functions as *cis*‐ or *trans*‐regulators of gene expression (Statello et al. 2021). The identification of lncRNAs typically involves RNA‐seq assembly, coding potential assessment (CPC/Pfam/CNPI/CAPT), and experimental validation (Uszczynska‐Ratajczak et al. 2018). For example, RNA sequencing has identified potential lncRNAs in *Vitis vinifera* that respond to the necrotrophic fungus *Botrytis cinerea* (Bhatia et al. 2021). Additionally, RNA sequencing of grape leaves of *Vitis piasezkii* ‘Liuba‐8’ and *Vitis vinifera* Pinot Noir identified an lncRNA, MSTRG.12742.1, which allegedly may play a positive role in grape resistance to downy mildew (Li et al. 2022). Transcriptome sequencing has identified *Vitis vinifera* lncRNAs responsive to powdery and downy mildews, with lncRNA‐coding sequence (CDS) pairs implicating their roles in the PTI immunity, including Ca^2+^ signalling, reactive oxygen species (ROS) metabolism, and pathogenesis‐related (PR) protein accumulation (Bhatia et al. 2021). Notably, miR827a has been shown to regulate stilbene synthesis by targeting *VqMYB14* and giving rise to susceptibility in grapevine immunity (Luo et al. 2024).

N6‐methyladenosine (m^6^A) is a modification resulting from methyl substitution on the sixth nitrogen (N6) atom on RNA adenosine, regulated by m^6^A recognising proteins (readers), methyltransferases (writers), and de‐methyltransferases (erasers) (Yue et al. 2019). m^6^A modification has been proved to play critical roles in regulating abiotic and biotic stress responses (Zhao, Han, et al. 2024). For instance, m^6^A methylation sequencing analysis of peanuts during infection with *Ralstonia solanacearum* revealed that m^6^A‐associated genes are involved in plant‐pathogen interactions, with *AhALKBH15* identified as an m^6^A demethylase that reduces m^6^A levels and upregulates the resistance gene *AhCQ2G6Y* (Zhao, Li et al. 2024). Similarly, *Malus hupehensis* YT521‐B homology domain‐containing protein 2 (*MhYTP2*) negatively modulates apple *Glomerella* leaf spot resistance by degrading *MdRGA2L* (Guo et al. 2023). However, how ncRNAs functionally intersect with RNA modifications like m^6^A to regulate grape immunity remains poorly understood.

Previously, we reported a specific genotype of the Chinese wild grape, *V. quinquangularis*, that shows strong aluminium tolerance and disease resistance (Luo et al. 2024; Jiang et al. 2023). Therefore, in this study, we analysed changes in non‐coding RNAs in two grape accessions (*V. quinquangularis* ‘Shanyang’ (SY) and *V. vinifera* ‘Cabernet Sauvignon’ (CS)) following flg22 treatment, as well as dynamic m^6^A modification in *V. quinquangularis* in response to treatment with flg22. We identified 150 miRNAs and 521 lncRNAs that responded to flg22. Subsequently, co‐expression analysis‐driven functional annotation revealed that miRNAs target genes were primarily enriched in ‘phenylpropane metabolism’ related pathways and ‘plant hormone signal transduction pathways’, while lncRNA *cis*‐target genes were associated with ‘stilbenoid, diarylheptanoid and gingerol biosynthesis’ and ‘plant‐pathogen interaction’. Additionally, we characterised the m^6^A methylome in ‘Shanyang’ and identified genes that showed significant correlations with m^6^A modifications. Integration of whole‐transcriptome and m^6^A methylation data allowed us to identify a *cis‐*target gene with the m^6^A modification gene, *LRR‐RLK* (Vitvi10g04478), whose transient expression improved grapevine resistance to *C. diplodiella*. Our findings provide new insights into the roles of m^6^A modifications and non‐coding RNAs in mediating plant‐flg22 interactions in grapevines.

## 1 Results

### 1.1 Genome‐Wide Identification and Characterisation of Non‐Coding RNAs in Grapevine Leaves Responding to flg22

**FIGURE 1:**
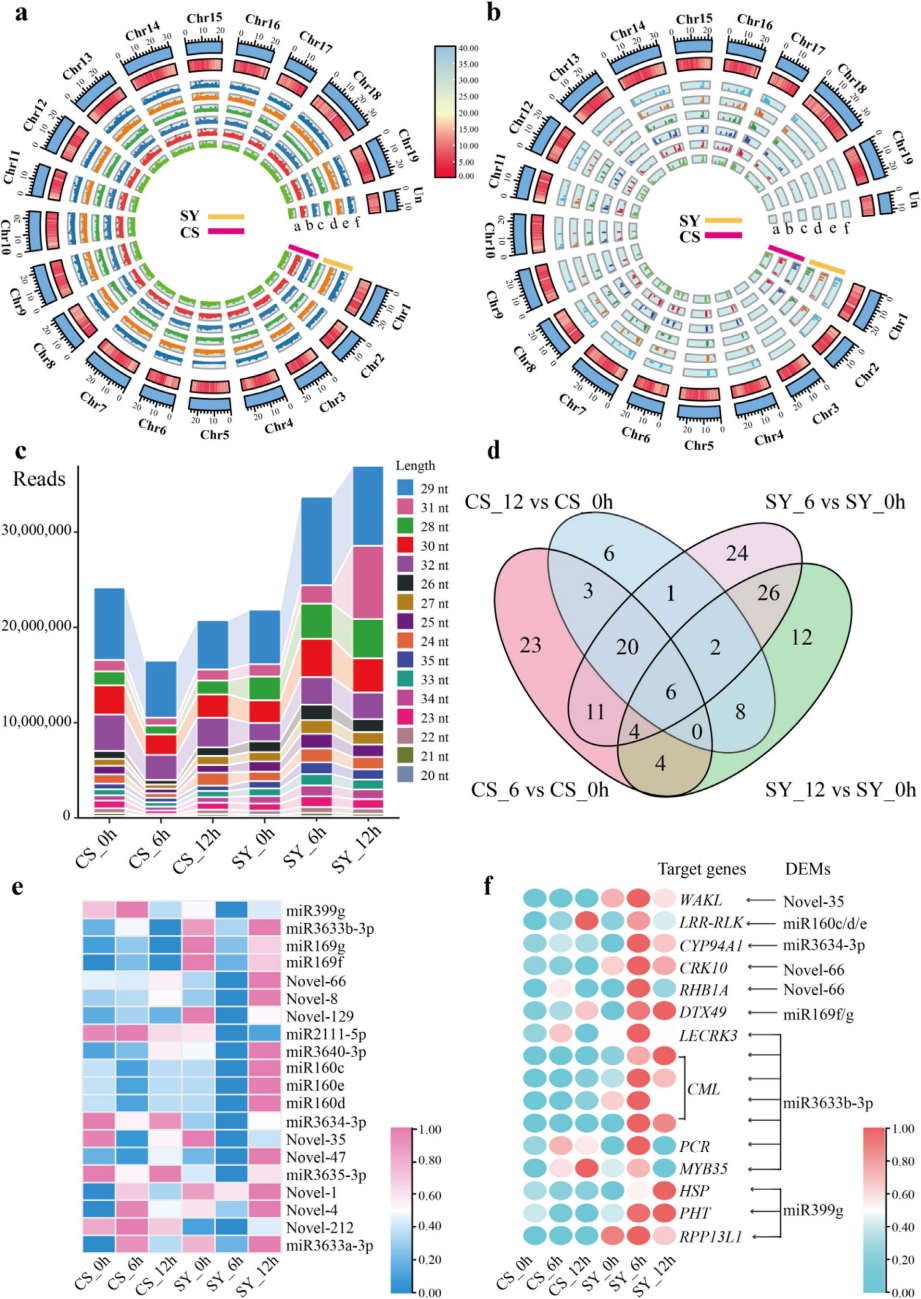
Analysis of ncRNA features and differentially expressed miRNA following flg22 treatment across different samples. (a) Tracks a–f correspond to lncRNAs identified in CS and SY from samples including CS_0 h, CS_6 h, CS_12 h, SY_0 h, SY_6 h and SY_12 h, respectively. (b) Tracks a‐f represent miRNAs identified in CS and SY from samples comprising CS_0 h, CS_6 h, CS_12 h, SY_0 h, SY_6 h and SY_12 h, respectively. The first track was the 19 chromosomes (Chr1‐Chr19) and the second track displayed gene density of the *V. vinifera* grape genome. The remaining tracks, labelled a–f, illustrate log_10_‐enrichment of counts per million (CPM), with values on Y‐axis derived from all samples in section a and b. The Y‐axis of lncRNA (tracks a–f) is set at 0–4.5, while that of miRNA is set at 0–6. (c) Columnar accumulation diagram displaying the distribution of small RNAs categorised by the lengths. (d) Venn diagram for DEMs in SY_6 vs. SY_0 h, SY_12 vs. SY_0 h, CS_6 vs. CS_0 h and CS_12 vs. CS_0 h groups. (e, f) Heat map of differentially expressed miRNAs and target genes of these differentially expressed miRNAs. *CML* (Vitvi01g01584, Vitvi01g02228, Vitvi01g02230, Vitvi01g02234), calcium‐binding protein; *CRK10* (Vitvi00g04597), cysteine‐rich receptor‐like protein kinase 10; *CYP94A1* (Vitvi07g00012), cytochrome P450 94A1; *DTX49* (Vitvi02g00403), protein DETOXIFICATION 49; *HSP* (Vitvi13g00491), 18.2 kDa class I heat shock protein; *LECRK3* (Vitvi13g02549), G‐type lectin S‐receptor‐like serine/threonine‐protein kinase LECRK3; *LRR‐RLK* (Vitvi08g02069), LRR receptor‐like serine/threonine‐protein kinase; *MYB35* (Vitvi14g01845), transcription factor MYB35; PCR (Vitvi01g00465), protein PLANT CADMIUM RESISTANCE 2; *PHT* (Vitvi07g00594), Inorganic phosphate transporter; *RHB1A* (Vitvi11g04256), E3 ubiquitin protein ligase RHB1A; *RPP13L1* (Vitvi13g02459), disease resistance RPP13‐like protein 1; *WAKL* (Vitvi17g00397), wall‐associated receptor kinase‐like. The arrow indicates the targeting relation. Heat map was plotted with FPKM values and averaged over three biological replicates for each sample.

To investigate the miRNA and lncRNA responses to flg22 treatment in grapevines, we generated approximately 47.55 million and 14.86 billion high‐quality sequencing reads from *Vitis quinquangularis* ‘Shanyang’ (SY) and *Vitis vinifera* ‘Cabernet Sauvignon’ (CS) leaves using miRNA‐seq and lncRNA‐seq techniques, respectively (Tables S1 and S2). The genomic distribution of the identified ncRNAs is illustrated in Figure 1a,b and detailed information is provided in Table S3. As shown in Figure 1a, lncRNA‐targeted regulatory genes were evenly distributed across the 19 chromosomes, with a broad distribution of *cis*‐ and *trans*‐regulatory elements. Notably, miRNAs were also distributed across all 19 chromosomes, but showed variable transcriptional levels across different chromosomal regions. Furthermore, except for chromosomes 3 and 19, miRNA‐targeted regulatory genes were widely detected throughout the grape genome (Figure 1b). Furthermore, the clean reads were obtained through small RNA (sRNA) sequencing representing 98.44% of the total raw reads (Table S1). Among these, 89 818 081 clean reads were successfully mapped to miRNA (known and novel), transfer RNA (tRNA), ribosomal RNA (rRNA), small nucleolar RNA (snoRNA) and small nuclear RNA (snRNA) (Table S4). For subsequent analysis, we focused on sRNA within the 20–35 nt length range. Length distribution analysis revealed that 24 nt sRNAs represented the most abundant group (Figure 1c).

### 1.2 The flg22 Treatment Elicited Time‐Dependent and Variety‐Specific Differential Expression Patterns of miRNAs in Grapevine Leaves

Analysis of pre‐miRNA sequences and identified 150 differentially expressed miRNAs (DEMs, *p*‐value < 0.05; Figure 1d). Further, differential analysis revealed 94 and 62 DEMs in SY at 6 and 12 h after treatment, respectively, compared with 0 h. In contrast, CS samples showed fewer DEMs (71 at CS 6 h and 46 at CS 12 h) (Figures S1 and 1d). Notably, we observed distinct temporal expression patterns between varieties, with several miRNAs, including miR3634‐3p, miR160c and Novel‐35, showing down‐regulation in SY_6 h, and lower expression levels at 6 h after treatment, compared to those in CS (Figures 1e and S2). Target‐genes prediction analysis revealed a higher expression of resistance‐related genes in SY than in CS at 6 h, including *LRR*‐*RLK*s, calmodulin protein (*CML*), and *MYB35* (Figure 1f). Interestingly, some miRNAs showed opposite expression trends in the varieties, such as miR399g, miR3633b‐3p and Novel‐66, which were down‐regulated in SY but upregulated in CS at 6 h after tlg22 treatment. Validation experiments confirmed the expression patterns of six significantly DEMs, which were consistent with the sequencing results (Figures S2 and 2a).

**FIGURE 2:**
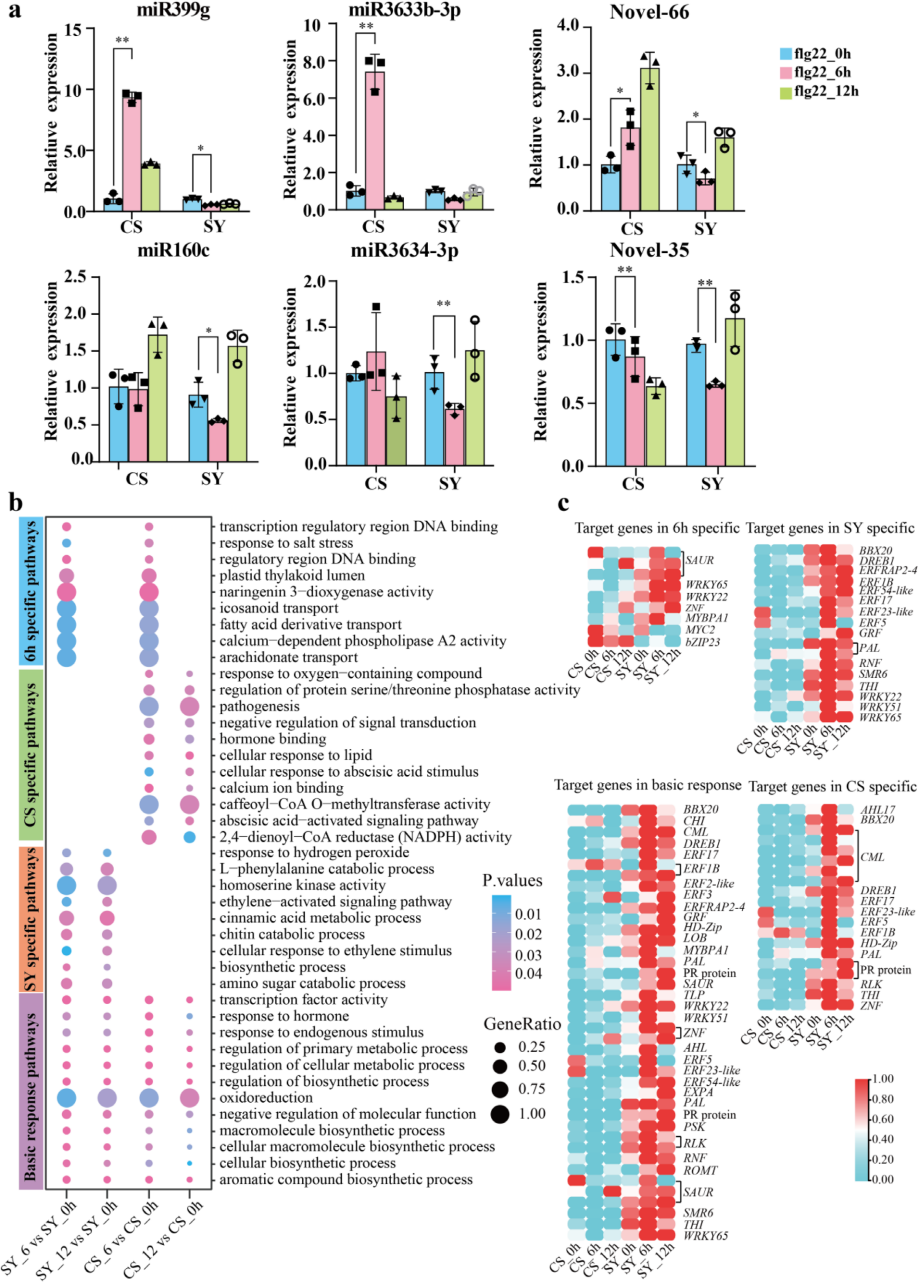
Analysis of differentially expressed miRNAs and functional enrichment of the target genes of miRNAs. (a) Expression pattern of miR399g, miR3633b‐3p, Novel‐66, miR3634‐3p, miR160c and Novel‐35 verified by RT‐qPCR (* (*p* < 0.05) and ** (*p* < 0.01)). The bars indicate the means ± standard error (SE) and average values and standard errors were derived from three biological replicates. (b) GO analysis of the target genes for differentially expressed miRNAs in four groups. The bar presented in purple, blue, green and orange was for different enrichment GO terms related to basic response, 6 h‐specific, CS‐specific and SY‐specific, respectively. (c) Heat map of targets genes for miRNAs on the right were involved in basic response, SY‐specific, CS‐specific and 6 h‐specific, respectively. Heat map was plotted with FPKM values and averaged over three biological replicates for each sample. *AHL17*, AT‐hook motif nuclear‐localised protein 17; *BBX20*, B‐box zinc finger protein 20; *bZIP23*, basic leucine zipper 23; *CHI*, chalcone flavanone isomerase; *DREB1*, dehydration‐responsive element‐binding protein 1E; *ERF1B*/*2‐like*/*3*/*5/17/23‐like/54‐like/RAP2‐4*, ethylene responsive transcription factor ERF1B/2‐like/3/5/17/23‐like/54‐like/RAP2‐4; *EXPA*, expansin‐like B1; *GRF*, growth‐regulating factor; *HD‐Zip*, homeobox leucine zipper protein; *LOB*, LOB domain‐containing protein; *MYBPA1*, MYBPA1 protein; *MYC2*, transcription factor MYC2; *PAL*, phenylalanine ammonia‐lyase; PR protein, major allergen pruav1; *PSK*, phytosulfokines; *RLK*, receptor‐like protein kinase; *RNF*, E3 ubiquitin protein ligase RNF; *ROMT,* trans‐resveratrol di‐O‐methyltransferase‐like; *SAUR*, auxin‐responsive protein SAUR; *SMR6*, cyclin‐dependent protein kinase inhibitor SMR6; *THI*, thiamine thiazole synthase; *TLP*, thaumatin‐like protein; *WRKY22*/*51*/*65*, WRKY transcription factor 22/51/65; *ZNF*, zinc finger protein. Abbreviations not mentioned in the figure legend can be found in the locations described earlier, such as in the legend of Figure 1f.

Gene Ontology (GO) functional annotation revealed that DEM target genes were enriched in basal response pathways, including transcription factor activity (GO:0003700), response to hormone (GO:0009725), and response to endogenous stimulus (GO:0009719) (Figure 2b; Table S5). Among 140 identified resistance‐related target genes, most encoded transcription factors (TFs), with ERF TFs being the most prevalent, followed by bZIP, NAC, WRKY, and bHLH TFs (Table S6); notably, 40 genes encoding pathogenesis‐related protein (PR), TFs, or receptor kinase genes showed higher expression levels in SY_6 h than CS_6 h, with up‐regulation in SY from 0 to 6 h (Table S6). And, 12 TF‐encoding genes (*MYC2*, DOF and HD‐Zip TFs) exhibited higher expression in CS_6 h than SY_6 h (Table S6).

Variety‐specific GO term analysis revealed SY‐specific (Go terms in SY_6 h vs. SY_0 and SY_12 h vs. SY_0 h) enrichment in phenylalanine ammonia‐lyase activity (GO:0045548) (Table S5) and ethylene‐activated signalling pathway (GO:0009873) (Figure 2b), with 48 TF‐encoding genes, mainly ERF or WRKY TFs (Table S6). In contrast, CS‐specific (Go terms in CS_6 h vs. CS_0 h and CS_12 h vs. CS_0 h) pathways include calcium ion binding (GO:0005509), negative regulation of signal transduction (GO:0009968), and pathogenesis (GO:0009405), predominantly involving calcium‐binding proteins, NAC and ERF TFs (Figure 2c; Table S6). Common pathways at 6 h‐specific (Go terms in SY_6 h vs. SY_0 h and CS_6 h vs. CS_0 h) included naringenin 3‐dioxygenase activity (GO:0045486) and fatty acid derivative transport (GO:1901571), with 25 TF‐encoding genes (Figure 2c; Table S6).

Kyoto Encyclopedia of Genes and Genomes (KEGG) pathway analysis identified significant enrichment in 15 pathways (Table S7; Figure S3). SY samples showed substantial enrichment in plant hormone signal transduction (ko04075) and phenylpropanoid biosynthesis pathways (ko00940) (*p* < 0.05) (Figure S3a,b), along with stilbenoid, diarylheptanoid, and gingerol biosynthesis pathway (ko00945), glutathione metabolism (ko00480) and MAPK signalling pathway (ko04016). Key genes in these pathways (SAUR, *ERF1*, *WRKY22*, *CML*, *GST*, *PAL*) showed higher expression in SY_6 h than CS_6 h (Figure S3e). CS samples were enriched in glutathione metabolism (ko00480) and plant pathogen interaction pathways (ko04626) (*p* < 0.05) (Figure S3c,d), with lower expression of CMLs and GSTs compared to SY (Figure S3e).

### 1.3 Characterisation and Analysis of Long Non‐Coding RNAs in Grapevine Leaves

**FIGURE 3:**
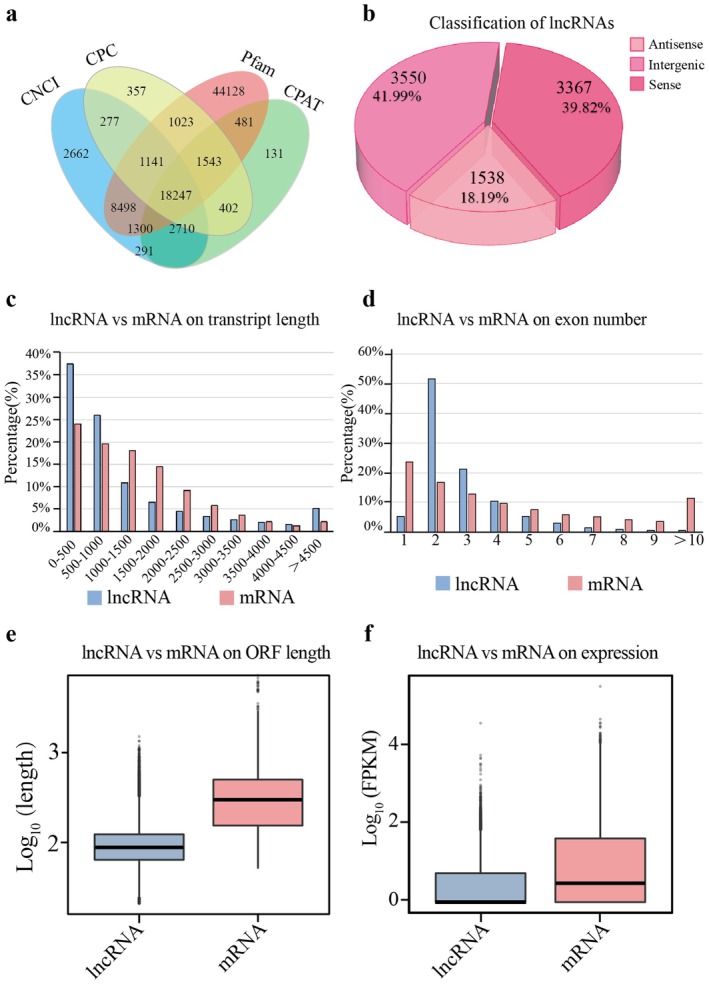
Identification of lncRNA features and differentially expressed lncRNA following flg22 treatment across all samples. (a) Venn diagram for identified lncRNAs with different software in all samples. (b) Pie diagram of percentage for lncRNAs types. (c, d) Chart diagrams of comparison in length and the exon numbers for mRNAs and lncRNAs. (e, f) Box diagrams of comparison in ORF length and expression for lncRNAs and mRNAs.

**FIGURE 4:**
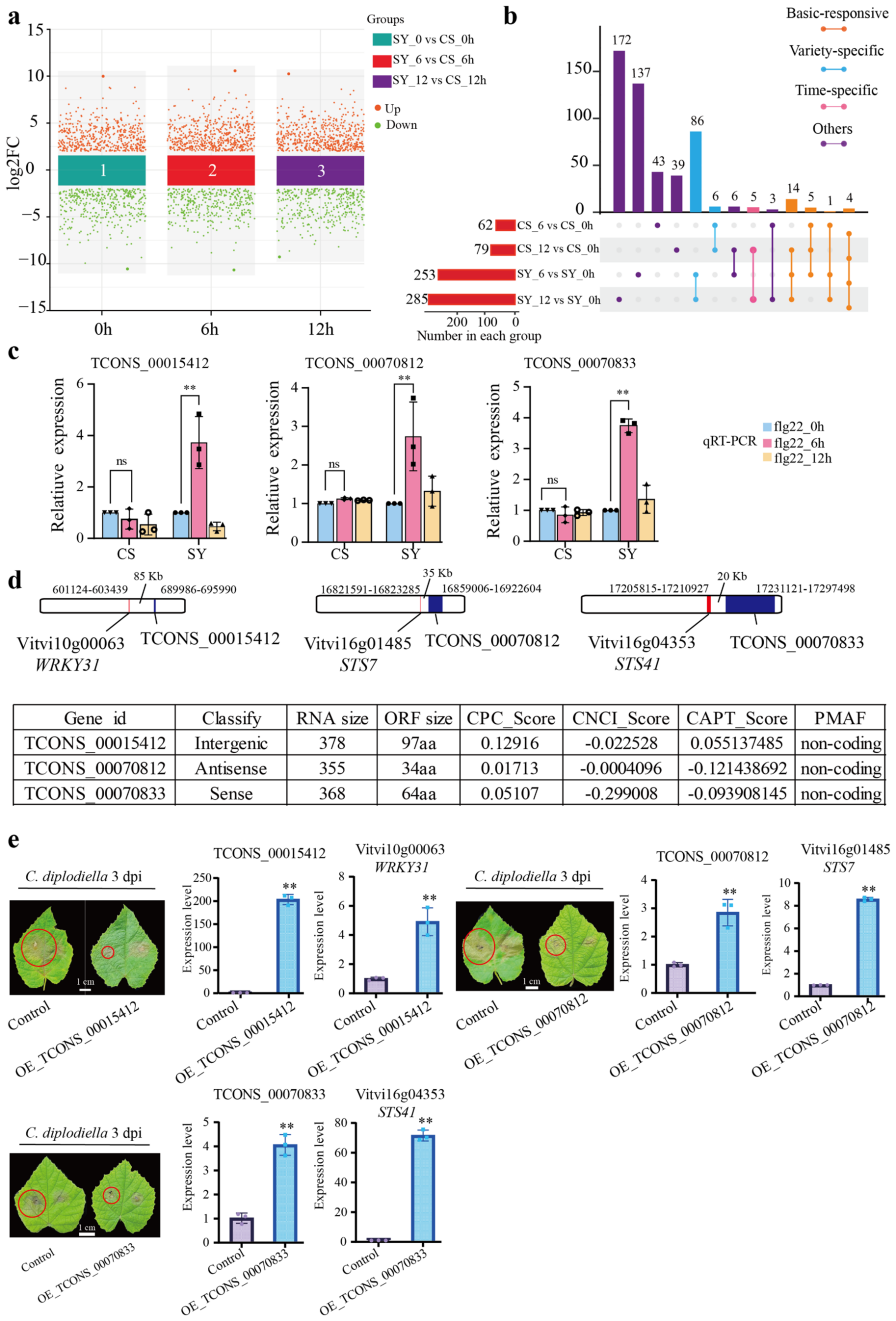
Identification of differentially expressed lncRNAs in different samples. (a) Differential analysis showing up‐ and down‐regulated lncRNAs across all three clusters. (b) Upset diagram analysis of DELs in SY_6 vs. SY_0 h, SY_12 vs. SY_0 h, CS_6 vs. CS_0 h and CS_12 vs. CS_0 h groups. (c) The expression pattern of differentially expressed lncRNAs verified by RT‐qPCR. * (*p* < 0.05) and ** (*p* < 0.01) display the levels of significant difference. The error bars represent the SE of three independent experiments. Values are means ± SE. (d) Schematic diagram of the location of lncRNAs and *cis*‐target genes on the chromosome and analysis of the coding potential by using different softwares. (e) The disease symptom on *V. quinquangularis* leaves temporarily overexpressing TCONS_00015412, TCONS_00070812, TCONS_00070833 (OE‐TCONS_00015412, OE‐TCONS_00070812 and OE‐TCONS_00070833) and control (Control: Non‐transgenic *V. quinquangularis*) in response to *C. diplodiella* infection. And the expression level of lncRNAs and their *cis*‐targets verified by RT‐qPCR. ** (*p* < 0.01) display the levels of significant difference. The error bars represent the SE of three independent experiments. Values are means ± SE.

Clean reads obtained from lncRNA‐seq showed high mapping rates, ranging from 98.03% to 98.70% (Table S8). Further, identified lncRNAs were classified as intergenic lncRNAs, antisense lncRNAs, or sense lncRNAs through comprehensive analysis (Table S9; Figure 3a,b). Most lncRNAs (63.44%) were located within 1000 bp of coding genes (Figure 3c,d; Table S10) and showed shorter open reading frames (ORFs) and lower expression than mRNAs (Figure 3e,f). Subsequently, we conducted a detailed examination of both induction profiles and quantitative changes to elucidate the expression dynamics of lncRNAs in response to flg22 treatment (Figure 4a). Differential expression analysis identified 253 and 285 DELs in SY at 6 and 12 h, respectively, compared to 0 h (Figure 4b). Further, at both 6 and 12 h after flg22, the number of upregulated lncRNAs exceeded that of downregulated lncRNAs in SY (Figure S4). In contrast, fewer DELs were observed in the CS grape, with 62 and 79 DELs identified at 6 and 12 h, respectively, compared to 0 h after flg22 treatment, respectively (Figure S4). A cross‐variety comparison revealed 21 DELs that were upregulated in both varieties, although with a greater fold changes in SY at 6 h after flg22 treatment (Figure S5c); meanwhile, *cis*‐target gene prediction for these DELs identified several receptor‐like kinases (*RLK*s) and transcription factors (TFs) showing higher expression levels in SY than in CS at 6 h after flg22 treatment. Additionally, we identified 10 DELs that displayed distinct expression patterns between the two varieties, showing significant upregulation in SY but downregulation in CS at 6 h after flg22 treatment. The *cis*‐target genes of the 10 DELs function as *RLK*s, *MAPKKK*, and disease resistance proteins (such as *RPP13L4* and *RIN4*) demonstrated more responsive expression in SY than in CS at 6 h after flg22 treatment (Figure S5c). Analysis identified seven differentially expressed lncRNAs that showed expression correlated with their *cis*‐target defence‐related genes, validated by RT‐qPCR (Figures 4c and S5a,b), suggesting their immune regulatory roles.

Further, we predict the potential coding ability of these lncRNAs. The result showed that TCONS_00015412, TCONS_00070812, and TCONS_00070833 could act through intergenic, antisense, and sense regulation, respectively. Results of the coding ability analysis for all lncRNA showed that TCONS_00015412, TCONS_00070812, and TCONS_00070833 do not seem to have the ability to encode proteins (Figure 4d). Then, we further verified the functions of lncRNAs. We constructed overexpression vectors for lncRNAs and transiently transformed the leaves of Chinese wild *V. quinquangularis*. The results showed that compared to the control group, overexpression of TCONS_00015412, TCONS_00070812, and TCONS_00070833 significantly upregulated the expression levels of their *cis*‐target genes *WRKY31*, *STS7*, and *STS41* (Figure 4e). Additionally, the infected leaf area was smaller in the overexpression group, indicating that TCONS_00015412, TCONS_00070812, and TCONS_00070833 play important roles in enhancing disease resistance in grapevines (Figure 4e).

### 1.4 Functional Analysis of Differentially Expressed lncRNAs

**FIGURE 5:**
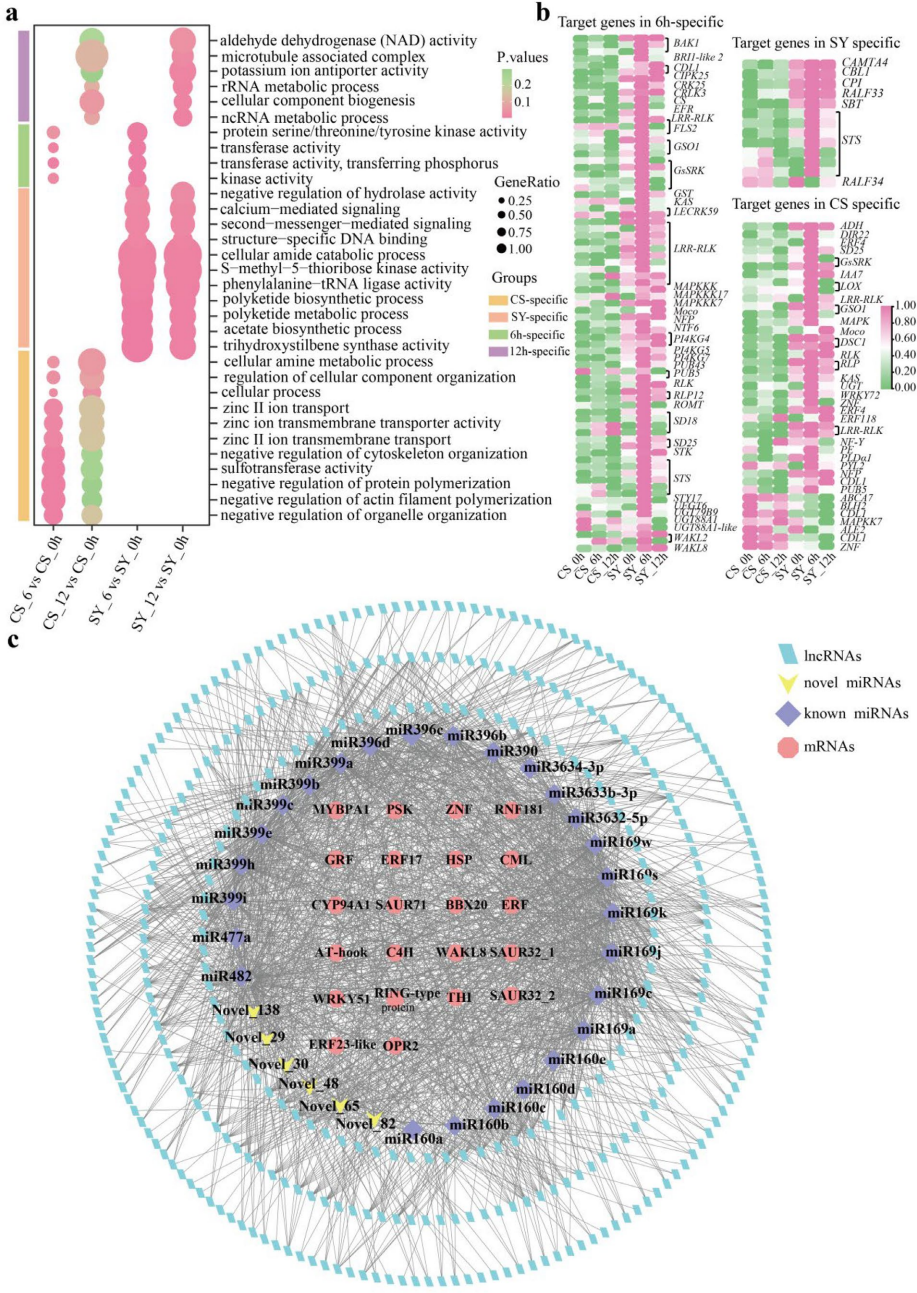
Functional enrichment analysis for *cis*‐target genes and the defence related ceRNA regulatory network in grapevine. (a) GO enrichment analysis of target genes for DE‐lncRNAs in different groups. (b) Heat map of target genes for lncRNAs in different GO terms group. *ABCA7*, ABC transporter A family member 7; *ADH*, alcohol dehydrogenase‐like 1; *ALE2*, receptor‐like serine/threonine‐protein kinase ALE2; *BLH2*, BEL1‐like homeodomain protein 2; *CAMTA4*, calmodulin‐binding transcription activator 4; *CDL1/BRI1‐like2/STK/STY17*, serine/threonine‐protein kinase CDL1/BRI1‐like2/STK/STY17; *CIPK25*, CBL‐interacting serine/threonine protein kinase 25; *CPI*, cysteine proteinase inhibitor 5‐like; *CS,* callose synthase; *DIR22*, dirigent protein 22; *DSC1*, Disease resistance‐like protein DSC1; *GSO1*/*EFR*, LRR receptor‐like serine/threonine‐protein kinase GSO1/EFR; *IAA7*, auxin‐responsive protein IAA7; *KAS*, 3‐ketoacyl‐CoA synthase; *LOX*, linoleate 13S lipoxygenase 2–1; *MAPKKK7*/*MAPKKK17*, mitogen‐activated protein kinase kinase kinase MAPKKK7/MAPKKK17; *Moco*, molybdenum cofactor sulfurase; *NFP*, serine/threonine receptor‐like kinase NFP; *NF‐Y*, nuclear transcription factor Y subunit C‐9; *NTF6*, mitogen‐activated protein kinase homologue NTF6; *PE*, pectinesterase; *PI4KG4/5/7*, phosphatidylinositol 4‐kinase gamma4/5/7; *PLDα1*, phospholipase D alpha 1; *PUB5*/*43*, U‐box domain‐containing protein 5/43; *PYL2*, abscisic acid receptor PYL2; *ROMT*, trans‐resveratrol di‐O‐methyltransferase‐like; *SD25*/*GsSRK*/*RLK*, G‐type lectin S‐receptor‐like serine/threonine protein kinase SD25/GsSRK/RLK; *UFGT6*, UDP‐glucose flavonoid 3‐O‐glucosyltransferase 6; *UGT79B9*/*88A1*, UDP‐glycosyltransferase 79B9/88A1. Abbreviations not mentioned in the figure legend can be found in the locations described earlier, such as in the legend of Figure 1 or the legend of Figure S5. (c) The ceRNA regulatory network in grapevine. The parallelogram, octagon, diamond shapes represent DELs, DEGs and known DEMs, respectively. ‘V’ form was novel DEMs. All the lines represent a targeting relationship among lncRNAs‐miRNAs‐mRNAs.

LncRNA primarily functions through *cis*‐regulation of neighbouring gene expression (Gil and Ulitsky 2020). To further elucidate the roles of flg22‐responsive differentially expressed lncRNAs (DELs), we analysed their target genes across different treatment groups (Table S11). GO enrichment analysis was performed to annotate the biological processes associated with the genes targeted by flg22‐responding DELs (Figure 5a; Table S12).

The results of GO enrichment annotation revealed that distinct functional categories existed between the SY and CS genotypes. Specifically, the enriched terms in SY at 6 and 12 h after flg22 treatment, relative to 0 h, were primarily related to trihydroxystilbene synthase activity (GO:0050350) or polyketide metabolic process (GO:0030638) (*p* < 0.05). Among these, *STS* genes were the most prevalent and followed by calmodulin‐binding proteins (Figure 5b; Table S13). Notably, *STS* genes involved in the trihydroxystilbene synthase activity pathway were up‐regulated in both SY and CS at 6 h after flg22 treatment, although the fold change was significantly higher in SY_6 h (Figure 5b). In contrast, CS‐specific GO pathways at 6 and 12 after flg22 treatment, were associated with zinc‐ion transmembrane transporter activity (GO:0005385), etc. Further, a total of 105 resistance‐related target genes were identified in CS‐specific GO pathways, predominantly encoding receptor‐like kinases/proteins (RLKs/RLPs), protein kinases, various transcription factors (TFs) and other resistance‐related genes (Table S13); most of which, interestingly, showed higher expression levels in SY than in CS at 6 h after flg22 treatment (Figure 5b). As for pathways that were common to SY and CS at 6 h relative to 0 h (6 h‐specific), the enriched terms included kinase activity (GO:0016301) and protein serine/threonine/tyrosine kinase activity (GO:0004712). Among the 163 genes enriched in these 6 h‐specific GO terms, RLKs were the most abundant, followed by protein kinases, UDP‐glycosyltransferase, and STS (Table S13). Notably, many RLKs, protein kinases, and STS showed higher expression levels in SY than in CS at 6 h after flg22 treatment (Figure 5b).

KEGG pathway analysis further highlighted the functional roles of *cis*‐target genes. In the SY_6 h vs. SY_0 h group, *cis*‐target genes were significantly enriched in the stilbenoid, diarylheptanoid, and gingerol (ko00945) pathway (Figure S6). In addition, the plant‐pathogen interaction (ko04626) was also enriched in this group. Analysis of the expression levels of these *cis*‐target genes in these pathways revealed that most *RLK*, *STS*s, and *ROMT* genes were upregulated in both SY_6 h and CS_6 h, but with greater fold changes in SY_6 h (Figure S6e). Furthermore, genes related to the plant‐pathogen interaction pathway, such as *CML* and disease resistance proteins, exhibited lower expression levels in CS_6 h compared to SY_6 h (Figure S6e). In the CS_6 h vs. CS_0 h group, *cis*‐target genes were also specifically analysed for their roles in defence response pathway. The results showed that respiratory burst oxidase (*Rboh*) and 12‐oxophytodienoic acid reductase (*OPR*) genes were upregulated at both CS_6 h and SY_6 h, with higher expression levels observed in SY_6 h compared to CS_6 h (Figure S6e).

### 1.5 Construction of Competitive Endogenous RNA Regulatory Networks

LncRNAs can indirectly regulate mRNAs expression levels by acting as competitive endogenous RNAs (ceRNAs) (Zhang et al. 2024). Given that differentially expressed miRNAs and lncRNAs exhibited a stronger response in the SY variety compared to CS, and considering that the 6 h treatment time point showed greater responsiveness to flg22 than the 12 h time point, we further investigated the plant defence‐associated ceRNA network. Specifically, differentially expressed genes and lncRNAs were predicted as potential targets of differentially expressed miRNAs, and a comprehensive ceRNA network involving DELs, DEMs and DEGs associated with plant defence/resistance was constructed (Figure 5c). Notably, this network included 22 resistance‐related genes or TFs, such as *CML*, *ERF*, *CYP94A1*, *WRKY51* and *MYBPA1*, which are known to play critical roles in plant defence. These genes formed a ceRNA network with 32 DEMs and 361 DELs (Figure 5c; Table S14).

### 1.6 m^6^A Modifications Occur Predominantly at Stop Codons, CDS and 3′UTR During *V. quinquangularis* Immunity Process

Based on these comprehensive whole‐transcriptome results, we found a more pronounced response in SY at 6 h after flg22 treatment. Consequently, we constructed m^6^A‐IP and RNA‐seq libraries of *V. quinquangularis* ‘Shanyang’ under flg22 treatment at 0 h and 6 h. Libraries yielded between 44 and 54 million reads (Table S15). The RNA‐seq exhibited a high degree of concordance across three independent biological replicates for each sample, thereby confirming the robustness and reliability of the obtained RNA‐seq data (Figure S7). Approximately 81.21%–85.10% of the clean reads were successfully aligned to the grape genome, with mapping to exon, intron, and intergenic regions (Figure S8; Table S16).

**FIGURE 6:**
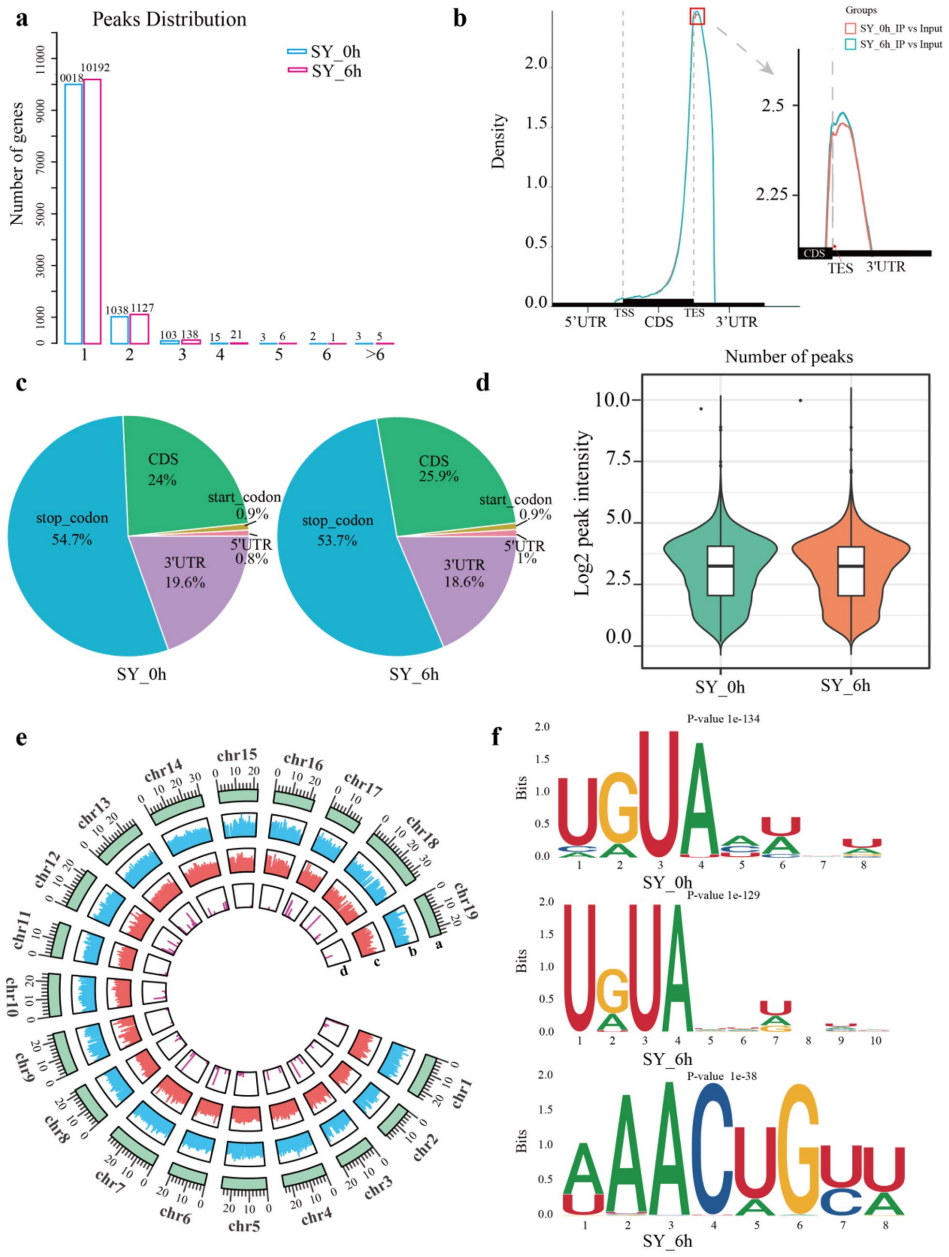
Effects of flg22 on m^6^A methylation of grapevine. (a) The number of genes with different m^6^A peaks. (b) The distribution of m^6^A peaks density among mRNA regions. TSS: Transcription start sites, TES: Transcription end sites. (c) Proportion of m^6^A peaks on functional elements (3′UTR, 5′UTR, CDS, start codon and stop codon) of genes after flg22 treatment. (d) Total m^6^A methylation levels during flg22 treatment. (e) The enrichment distribution of m^6^A peaks and gene expression on 19 chromosomes after flg22 treatment 0 and 6 h: (a) This track was the 19 chromosomes (Chr1‐Chr19), (b, c) Enrichment of peaks in immunoprecipitation library and input library after 0 and 6 h treatment with flg22, respectively, (d) log_2_(FC) of differential m^6^A modification peaks. (f) Sequence logo representing the most conserved motif URUAY and RRACH with m^6^A containing peaks (R = G/A, Y = A/C/G/U, H = A, C or U).

A total of 13 029 m^6^A peaks corresponding to 11 490 genes were identified in SY leaves after 6 h of flg22 treatment, whereas 12 521 peaks associated with 11 182 genes were identified in the same genotype at 0 h after flg22 treatment (Table S17). The peaks in SY at 0 h after flg22 treatment were distributed in the transcription start site (TSS) and transcription end site (TES) regions; however, a greater number of peaks were located in the TSS than in the TES region at 6 h after flg22 treatment in the same genotype (Figure S9). Notably, the number of transcripts containing one or more m^6^A peaks increased progressively when grape leaves were treated with flg22 for 6 h (Figure 6a). We divided the genes into five non‐overlapping regions to analyse the distribution pattern at the transcriptome level, including 3′UTR, 5′UTR, CDS, and start and stop codons, and analysed m^6^A peak‐density distribution (Figure 6b). Further analysis of the m^6^A enrichment ratio showed a slight decrease in the proportion of m^6^A peaks located in the 3′UTR and stop codon regions in SY from 0 to 6 h after flg22 treatment (Figure 6c). Overall, global methylation of m^6^A did not show significantly different changes in SY between 6 and 0 h after flg22 treatment (Figure 6d), and the distribution patterns of m^6^A peaks across grape chromosomes were relatively uniform (Figure 6e).

Additionally, m^6^A modification typically occurs within conserved sequence motifs in both plants and animals. We explored these conserved motifs in the grape transcriptome and identified the most conserved motif as ‘URUAY’ in both SY_0 h and SY_6 h (Figure 6f), which has also been reported in apple (Xu et al. 2023), tomato (Zhou et al. 2019) and *Arabidopsis* (Wei et al. 2018). Further analysis revealed that the m^6^A‐modified regions matching the URUAY motif in our results were predominantly UGUAC and UGUAU. Moreover, the most common RRACH motif in plants was exclusively found in SY_6 h, with the m^6^A‐modified region matching this motif being AAACU (Figure 6f).

### 1.7 Differentially Methylated Genes Caused by flg22 Were Enriched in Diverse Biological Processes

**FIGURE 7:**
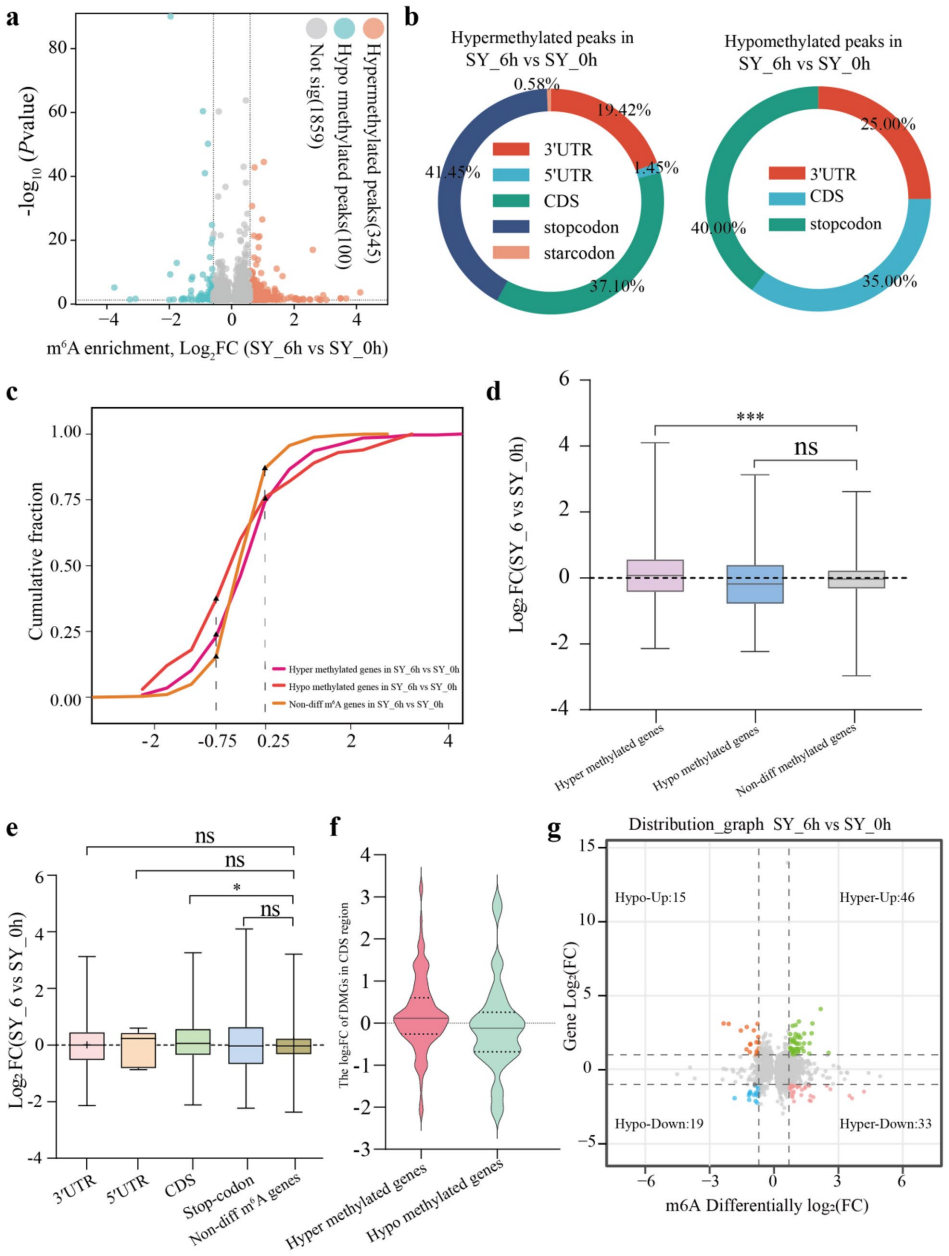
The relationship between mRNA abundance and m^6^A modification in grapevine. (a) The volcano diagrams of differential m^6^A peaks in SY_6 vs. SY_0 h. (b) Distribution of significantly differentially modified m^6^A peaks on functional elements of genes. (c) The cumulative distribution analysis of gene expression alterations among hyper‐ and hypo‐DMGs, and no‐differential m^6^A‐modified genes. The x‐axis represents the log_2_FC values of gene expression levels. (d) Box diagram of log_2_FC of hyper‐DMGs, hypo‐DMGs and no‐diff methylated genes. (e) The box diagram of DMGs expression changes among different gene features and non‐diff m^6^A‐modified genes in SY_6 vs. SY_0 h. * (*p* < 0.05) and *** (*p* < 0.001) display the levels of significant difference. (f) Violin diagram of of log_2_FC of hyper‐DMGs, hypo‐DMGs in CDS region. (g) Correlation between DMGs and DEGs expression. Orange dots (Hypo‐up): hypomethylation and up‐regulated genes; blue dot (Hypo‐down): hypomethylation and down‐regulated genes; green dots (Hyper‐up): hypermethylation and up‐regulated genes; pink dots (Hyper‐down): hypermethylation and down‐regulated genes.

We identified significantly differentially methylated peaks (DMPs) based on *p*‐value < 0.05 and a fold change ≥ 1.5. Specifically, the results revealed 100 hypomethylated and 345 hypermethylated DMPs (Figure 7a), corresponding to 99 and 336 transcripts (named as DMGs), respectively (Table S18). Hypermethylated peaks were mainly distributed around the stop codon (41.45%) and CDS (37.10%), and hypomethylated peaks were significantly enriched in the stop codon (40.00%) and CDS (35.00%) regions (Figure 7b).

To elucidate the functional implications of DMGs, we conducted GO and KEGG analyses (Figure S10). The GO annotation results revealed that hypermethylated genes were significantly (*p* < 0.05) enriched in immune‐related pathways, including cysteine biosynthetic process from serine (GO:0006535), protein kinase C‐activating G‐protein coupled receptor signalling pathway (GO:0007205), jasmonic acid metabolic process (GO:0009694) and salicylic acid metabolic process (GO:0009696) (Figure S10a; Table S19). Similarly, KEGG analysis revealed enrichment in pathways such as biosynthesis of secondary metabolites (ko01110) and plant‐pathogen interaction (ko04626) (Figure S10c). These findings suggest that m^6^A modifications may play a regulatory role in plant defence mechanisms.

To investigate the relationship between mRNA and m^6^A methylation levels during the grapevine immunity response, we analysed the expression levels of DMGs and evaluated the correlation between m^6^A changes and gene expression using cumulative distribution and boxplot analyses (Figure 7c,d). Cumulative distribution analysis showed that the expression levels of DMGs, including hypomethylated and hypermethylated genes, were lower than those of non‐differential m^6^A genes when log_2_FC < −0.75, whereas they were higher when log_2_FC > 0.25 (Figure 7c). Boxplot analysis revealed that hypermethylated DMGs showed significantly higher expression levels in SY at 6 than at 0 h of flg22 treatment, whereas hypomethylated DMGs showed a slight decrease at 6 h. In contrast, the non‐differential m^6^A‐modified genes did not exhibit this trend (Figure 7d). Additionally, considering the distribution patterns of hypermethylated and hypomethylated m^6^A peaks, which were primarily located in close proximity to the CDS, stop codon, or 3′UTR regions (Figure 7b), we categorised DMGs into CDS, 5′UTR, 3′UTR, and stop codon regions. Comparative analysis revealed that DMGs in the CDS region showed higher expression levels than non‐differential m^6^A genes, whereas no significant differences were observed in the 3′UTR, stop codon, or 5′UTR regions (Figure 7e). Notably, hypermethylated DMGs in the CDS region showed elevated expression levels (Figure 7f), suggesting that m^6^A modifications in the CDS region generally enhanced mRNA abundance.

Meanwhile, RNA‐seq analysis identified 1725 upregulated and 2714 downregulated differentially expressed genes, |log_2_FC| > 1 and *p*‐value < 0.05, in SY at 6 compared with 0 h after flg22 treatment (Figure S11a). Further comparison between m^6^A DMGs and DEGs revealed 113 DEMGs showing both differential m^6^A modification and differential expression (Figure S11b). Among these, 79 hypermethylated DEMGs included 46 upregulated and 33 downregulated genes, while 34 hypomethylated DEMGs comprised 15 upregulated and 19 downregulated genes (Figure 7g), indicating a complex relationship between m^6^A modification and gene expression.

In fact, GO analysis of DEMGs highlighted the enrichment of RNA‐related pathways, such as mRNA transcription (GO:0009299), tRNA modification (GO:0006400), and tRNA metabolic processes (GO:0006399) (Table S20). Additionally, DEMGs were enriched in pathways related to biotic and abiotic stress responses, including calcium ion binding (GO:0005509), jasmonic acid metabolic processes (GO:0009694), phenylpropanoid catabolic processes (GO:0046271), and hydroquinone: oxidoreductase activity (GO:0052716) (Figure S11c). KEGG analysis of DEMGs further indicated significant enrichment in pathways such as ABC transporters (ko02010), alpha‐linolenic acid metabolism (ko00592), and plant‐pathogen interactions (ko04626) (Figure S11d). These results underscore the widespread involvement of m6A modifications in the early immune response of *V. quinquangularis*.

**FIGURE 8:**
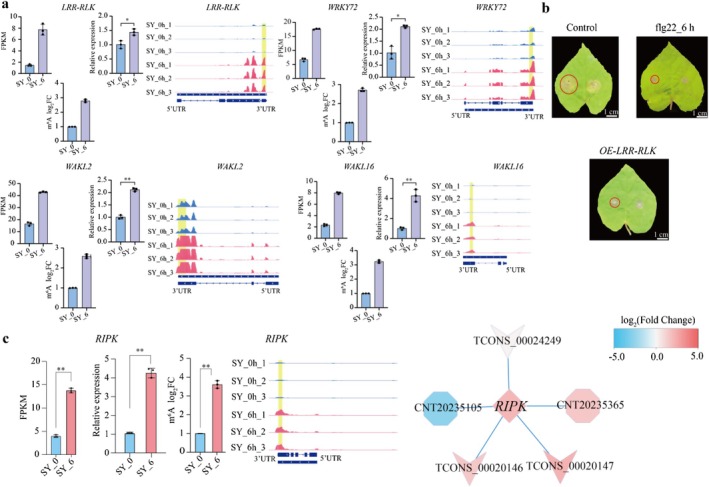
m^6^A modification level of defence related genes and its expression changes affect grape immunity. (a) The transcript levels of *LRR‐RLK* (Vitvi10g04478), *WRKY72* (Vitvi01g00940), *WAKL2* (Vitvi03g00882) and *WAKL16* (Vitvi18g02513) determined by RNA‐seq and RT‐qPCR; and the levels of m^6^A enrichment in grape at SY_0h and SY_6h. * (*p* < 0.05) and ** (*p* < 0.01) display the levels of significant difference. The error bars represent the SE of three biological experiments. Values are means ± SE. Integrative Genomics Viewer (IGV) tracks exhibiting the distribution of m^6^A peaks in mRNAs of the *LRR‐RLK*, *WRKY72*, *WAKL2* and *WAKL16*. The hypermethylated m^6^A peaks in SY_6 h sample compared with those at SY_0 h. Yellow shaded areas indicate the position of the methylation peaks. (b) The disease symptom on *V. quinquangularis* leaves temporarily overexpressing *LRR‐RLK* (OE‐*LRR‐RLK*), control (Non‐transgenic *V. quinquangularis*) and flg22 treatment for 6 h (flg22_6 h: Control sample was treated with 6 h) in response to *C. diplodiella* infection. (c) Transcript levels of *RIPK* determined by RNA‐seq and RT‐qPCR and m^6^A enrichment by m^6^A‐seq. The error bars represent the SE of three biological experiments. Values are means ± SE. IGV tracks showing the distribution of m^6^A reads in transcripts of RIPK; Predicted results of the lncRNA‐mRNA regulatory network. The octagon, ‘V’ and quadrangle shapes represent known DELs, novel DELs and mRNA, respectively. And the colour of different molecules represents log_2_FC in SY_6 vs. SY_0 h.

Flg22 is known to rapidly induce early response genes, including leucine‐rich repeat receptor‐like kinase genes (*LRR‐RLK*s), TFs and kinases. Our m^6^A‐seq analysis identified four genes exhibiting hypermethylation in the CDS or stop codon region under flg22 induction, including *LRR‐RLK*, *WAKL* (Wall‐associated receptor kinase‐like) and WRKY TF (Figure 8a). These genes, implicated in plant immunity and biotic stress responses (Li et al. 2020), showed significantly increased expression levels in SY_6 h compared to SY_0 h, as validated by RT‐qPCR (Figure 8a). Furthermore, transient overexpression of the *LRR‐RLK* gene (Vitvi10g04478) in *V*. *quinquangularis* leaves demonstrated significantly reduced lesion sizes upon *C. diplodiella* infection compared to the control (Figure 8b). Similarly, flg22‐treated leaves exhibited smaller lesion sizes than under controls. These findings suggest that *LRR‐RLK* (Vitvi10g04478) positively regulates grapevine immunity defence, enhancing resistance against *C. diplodiella*.

### 1.8 Combined Analysis of Non‐Coding RNAs and m^6^A Modification Genes in Grape Response to flg22

We further conducted an integrative analysis of target genes of ncRNAs and DEMGs, identifying one target gene exhibiting m^6^A modification (Figure 8c). As shown in Figure 8c, the IGV diagram shows hypermethylation of the serine/threonine‐protein kinase RIPK (*RIPK*) under the SY_6 h condition, with this gene showing up‐regulation in SY_6 h compared to SY_0 h. Additionally, the combined analysis revealed that three lncRNAs were predicted to positively regulate *RIPK* (Figure 8c).

## 2 Discussion

Most studies have used single‐ or multiple‐omics applications, including transcriptome, whole‐transcriptome, proteome, metabolome, and m^6^A methylation, to investigate plant responses to pathogen invasion or PAMPs challenges in plants such as *Arabidopsis* (Bjornson et al. 2021), tomato (Hu et al. 2022), rice (Tang et al. 2021), and peanut (Zhao, Li, et al. 2024). In this study, we used whole‐transcriptome sequencing and MeRIP‐seq to analyse flg22‐triggered PTI immunity in grapevines, providing new insights into the mechanisms underlying immunity in plants.

### 2.1 Differential Non‐Coding RNA Regulatory Networks Drive Genotype‐Specific Immune Responses to flg22 in Grape

Our study reveals distinct patterns of DEMs and DELs between SY and CS in response to flg22 treatment, suggesting genotype‐specific regulatory mechanisms in grapevine immunity. The more pronounced differential expression of ncRNAs in SY, particularly at 6 h post‐treatment (Figures 2a and 4c), aligns with emerging evidence that ncRNAs serve as key modulators of plant stress responses (Wang et al. 2017). The stronger flg22‐induced ncRNA expression in SY compared to CS further supports the activation of grape immunity in both genotypes, though with differing intensities, potentially reflecting genetic variation in immune signalling efficiency.

Notably, miR399g and miR3633b‐3p exhibited downregulation in SY post‐flg22 treatment (Figure 2a), concomitant with the upregulation of their target genes, including pathogenesis‐related (PR) proteins and calcium‐binding proteins (e.g., CML). This inverse correlation suggests their roles in fine‐tuning defence‐related gene expression, consistent with prior reports linking miR399 to ethylene‐mediated stress responses (Kang et al. 2014) and miR3633b‐3p to cold adaptation (Sun et al. 2015). And, the upregulation of miR160c and miR3634‐3p target genes (e.g., *LRR‐RLK*s and *CYP94A1*) in SY (Figure 1f) implies their involvement in reinforcing PAMP‐triggered immunity. miR160a, as a conserved miRNA, regulates callose deposition during PAMP‐triggered immune responses (Feng et al. 2022).

Furthermore, DEL‐associated *cis*‐target genes (e.g., RLKs, STS, ROMT, OPR and CML) were more highly expressed in SY (Figures S5c and S6e), implicating lncRNAs in the transcriptional regulation of defence pathways. The enhanced disease resistance observed upon transient overexpression of lncRNAs TCONS_00015412, TCONS_00070812, and TCONS_00070833 in *V. quinquangularis* (Figure 4d,e) underscores their functional significance. These lncRNAs may act as scaffolds, decoys, or epigenetic regulators to modulate immune gene expression, though their precise mechanisms warrant further investigation.

### 2.2 Enhanced and Sustained Defence Activation Underlies the Stronger flg22 Response in SY Grape

The observed genotype‐specific immune responses to flg22 in grapevine appear to stem from differential activation of pattern‐triggered immunity (PTI) components. Our data reveal that SY exhibits more robust and coordinated induction of multiple defence‐related pathways compared to CS, particularly at the critical 6 h post‐flg22 treatment time point (Figures 2c and 5b). The stronger upregulation of PRR‐related genes (including *BAK1*, *LRR‐RLK*s, *WAKL*s) in SY (Figure 5b) suggests more efficient perception and initial signal transduction of flg22. This is particularly noteworthy given that *BAK1* serves as a central regulatory hub for multiple PRR complexes and *WAKL*s have been shown to integrate cell wall integrity monitoring with immune signalling, such as *CsWAKL08* (Li et al. 2020). The coordinated upregulation of these receptors in SY may create a stronger initial immune response. In this study, differential TF activation patterns between genotypes reveal potential regulatory mechanisms underlying SY's enhanced immunity. ERF genes, including *ERF2*‐like, *ERF3*, *ERF4*, and *ERF23‐like*, were expressed at significantly higher levels in SY than CS at 6 h after flg22 treatment (Figures 2c and 5b), mirroring findings in other studies where these TFs regulate phytoalexin production and bacterial resistance (Song et al. 2019; Shi et al. 2022). *ERF23* plays a crucial role in the early‐stage response of wheat to *Puccinia striiformis f. sp. tritici* (*Pst*) (Ren et al. 2024).

Studies have shown that *SlWRKY22* enhances immunity in both tomatoes (Ramos et al. 2023) and *Nicotiana benthamiana* (Ramos et al. 2021), conferring resistance against *Pst*. Similarly, *PIWRKY65* positively regulates SA‐ and JA‐mediated resistance in *Paeonia lactiflora* (herbaceous peony) (Wang et al. 2020). Furthermore, *AtWRKY51* regulates salicylic acid‐mediated signal transduction pathways during plant immunity (Gao et al. 2011). In this study, the elevated expression of *WRKY22*, *WRKY65*, and *WRKY51* in SY may correlate with their established roles in SA/JA‐mediated resistance. Additionally, the reduced *MYC2* expression in SY aligns with its role as a negative regulator of resistance to *B. cinerea* infection (Zhai et al. 2013). This may indicate strategic suppression of JA responses during early PTI activation in SY.

The production of ROS is regulated by the respiratory burst oxidase (*Rboh*) and glutathione S‐transferase (*GST*). The stronger induction of Rboh and GST genes in SY suggests more potent ROS generation and management. This is consistent with *BIK1*‐mediated RbohD phosphorylation mechanisms (Kadota et al. 2014) and *GaGSTF9*‐mediated resistance to *Verticillium dahliae* in Chinese *Gossypium arboreum* (Gong et al. 2018). Moreover, genes associated with the stilbene synthesis pathways, including *PAL*, *STS*, and ROMT, were induced and expressed at higher levels in SY at 6 h post‐flg22 treatment, pointing to enhanced phenylpropanoid and stilbene production capacity in SY (Figures 2c and 5b). This metabolic reprogramming likely contributes to both immediate defence and longer‐term resistance. The more pronounced activation of CML and MAPK cascade components in SY suggests more efficient signal amplification. These pathways are known to integrate multiple defence signals (Jiao et al. 2016). In conclusion, the coordinated upregulation of these interconnected defence components in SY suggests that there is more efficient signal perception, transduction machinery, and a stronger metabolic response to defence in SY grape.

### 2.3 Dynamic m^6^A Methylation Regulates Immune Gene Expression in Grapevine Response to flg22

Our study reveals that m^6^A methylation serves as an important regulatory layer in grapevine immune responses to flg22 treatment. The observed changes in m^6^A modification patterns following pathogen elicitation suggest an epitranscriptomic mechanism that fine‐tunes defence‐related gene expression in SY grapevines. The detection of differential m^6^A methylation patterns in immune‐related genes after flg22 treatment provides compelling evidence for the involvement of RNA methylation in grapevine defence responses. Notably, our observation that hypermethylation in coding sequences (CDS) correlates with increased mRNA abundance (Figure 6e) aligns with recent findings in strawberry (Zhou et al. 2021) and suggests a conserved regulatory mechanism in perennial plants. This positive correlation between CDS methylation and transcript stability may represent an important strategy for rapid immune gene activation. Studies have shown that m^6^A modification is involved in modulating the expression of host immune‐related genes, which in turn influence immune responses. For example, In rice, study found that m^6^A modification levels exhibited dynamic changes during rice‐virus interactions, with a notable increase after viral infection (Zhang et al. 2021). Additionally, the *Malus hupehensis* gene *MhYTP*2 affects m^6^A modification of susceptibility gene *MdMLO19* during powdery mildew infection (Guo et al. 2022). Our work extends these findings to grapevine‐pathogen interactions, revealing both conserved and potentially unique aspects of epitranscriptomic regulation in woody perennials.

Additionally, we identified and validated several m^6^A‐modified immune genes, including *WRKY72*, *WAKL2*, *WAKL16* and *LRR‐RLK*. The flg22‐induced expression pattern of *WRKY72* mirrors its established role in bacterial defence (Bhattarai et al. 2010); and these cell wall‐associated kinases show methylation changes consistent with their signalling roles. Excitingly, transient overexpression of the *LRR‐RLK* (Vitvi10g04478) gene confirms its functional importance in disease resistance in *V. quinquangularis*. The preliminary success of *LRR‐RLK* (Vitvi10g04478) overexpression suggests that m^6^A‐modified immune genes represent promising targets for resistance breeding. These findings establish m^6^A methylation as a significant component of grapevine immune regulation and provide a foundation for understanding how epitranscriptomic modifications contribute to disease resistance in perennial crops.

## 3 Conclusion

In this study, we identified non‐coding RNAs involved in grapevine responses to flg22 treatment and characterised the m^6^A methylome in ‘Shanyang’. Co‐expression analysis revealed that the ncRNA target genes were mainly associated with defence responses. Notably, transient overexpression of lncRNAs TCONS_00015412, TCONS_00070812, and TCONS_00070833 in *V. quinquangularis* conferred significantly enhanced disease resistance compared to the control plants. Furthermore, a *cis*‐target gene with an m^6^A modification, *LRR‐RLK* (Vitvi10g04478), was confirmed to enhance grapevine resistance to *C. diplodiella*. These findings provide novel insights into the roles of m^6^A modifications and ncRNAs in mediating plant‐flg22 interactions, thereby advancing our understanding of grapevine immune response and offering potentially effective approaches for disease‐resistant breeding. However, the mechanisms by which m^6^A regulates the expression of disease resistance genes such as *LRR‐RLK* (Vitvi10g04478), as well as what specific writer/reader/eraser proteins mediate these immune‐related m^6^A changes, remain to be further elucidated. Additionally, the precise molecular mechanisms of key candidate lncRNAs and their functional contributions to disease resistance in grapevine represent critical focuses for future research.

## 4 Materials and Methods

### 4.1 Plant Materials and flg22 Treatment

Chinese wild grapevine *V. quinquangularis* ‘Shanyang’ (SY) leaves were collected from Shanyang County, Shangluo City, Shaanxi Province, China. Among the collected genotypes, the wild *Vitis* material used in this research was specifically labelled SY_5. European grapevine *V. vinifera* ‘Cabernet Sauvignon’ (CS) leaves were collected from the Life Science Experimental Park at Northwest University, Xi'an, Shaanxi, China. Fully expanded leaves, exhibiting a comparable developmental stage, were harvested from randomly selected individuals from the apex of SY and CS grapevines. These leaves were promptly transported to the laboratory and treated with 1 μM flg22 solution (GenScript). Subsequently, the leaves were frozen in liquid nitrogen and stored at −80°C. The flg22‐0 h treatment served as the control, with each group of samples having three biological replicates. The flg22 powder was dissolved using sterile water.

### 4.2 Extraction of Total RNA

Total RNA was extracted following the protocol of the Plant Total RNA kit (Omega‐Bio‐Tek, China) steps and detected by Agilent 2100 Bioanalyzer (Agilent Technologies, Palo Alto, CA, USA) and a NanoDrop Spectrophotometer (Thermo Scientific, Waltham, MA, USA). MeRIP‐seq and whole transcriptome sequencing were subsequently performed. The sequencing libraries for the whole transcriptome were abbreviated as SY_6 h or SY_12 h, CS_6 h or CS_12 h, representing samples treated with flg22 for 6 h or 12 h, respectively. SY_0 h or CS_0 h were used as parallel controls. SY_0 h and SY_6 h were also used as abbreviations for m^6^A sequencing libraries. All sequencing data in this study (including whole transcriptome‐seq and MeRIP‐seq) were generated with three independent biological replicates for each condition.

### 4.3 Library Construction and Sequencing for ncRNAs

Approximately 3 μg of total RNA per sample was utilised for miRNA sequencing. The Small RNA Sample Prep Kit was employed to generate sequencing libraries, using qualified total RNA for library construction. The quality of the libraries was assessed with an Agilent 2100 Bioanalyzer, and sequencing was conducted on the Illumina HiSeq 2500 platform. Raw reads containing adapters and low‐quality bases were removed to obtain clean reads. Clean reads with a length of 18–30 nt were screened and mapped on the reference grape genome ( *Vitis vinifera* PN40024.v4.53) using Bowtie 2 (Langmead and Salzberg 2012). The distribution and types of mapped sRNAs were further analysed: mapped reads were aligned with sRNAs in the Rfam databases, and tags matching rRNA, snoRNA, snRNA, and tRNA were discovered and removed. The sRNA was also compared to repeat sequences and to the introns and exons of mRNAs. Known miRNAs and novel miRNAs were identified using the miRbase and miREvo databases, respectively (Wen et al. 2012).

For lncRNA sequencing, approximately 5 μg of total RNA per sample was used. Ribosomal RNA and residual free rRNA were removed using the Epicentre Ribo‐zero rRNA removal kit (Epicentre, USA). Linear RNA was then eliminated with 3 U of RNase R (Epicentre, USA) per μg of RNA. Following the manufacturer's recommendations, sequencing libraries were generated using the NEBNext Ultra Directional RNA Library Prep Kit for Illumina (NEB, USA). The library quality was evaluated on the Agilent 2100 bioanalyzer, and sequencing was performed on the Illumina Hi‐seq 4000 platform. Transcripts with exon ≥ 2 and length > 200 bp were further analysed, and transcripts without coding potential predicted by the CPC, CNCI, CPAT, Pfam software (Finn et al. 2014), were candidate novel lncRNAs. The newly identified lncRNAs were categorised into lincRNA, intronic lncRNA, anti‐sense lncRNA, and sense‐overlapping lncRNA based on their positional relationship with known mRNA. Known lncRNAs were identified using the NONCODE and GreeNC databases.

### 4.4 Differential Expression Analysis of ncRNAs and mRNAs

The expression level of ncRNA were quantified using fragments per kilobase of transcript per million mapped reads (FPKM) values calculated by StringTie software, while miRNA expression levels were normalised using transcripts per million reads (TPM). Differentially expressed lncRNAs (DELs) and genes (DEGs) were identified based on the criteria of fold change (FC) > 2 and *p*‐value < 0.05 between the flg22 treatment and control groups. Differentially expressed miRNAs (DEMs) were selected with a *p*‐value < 0.05. The DESeq2 (version 1.12.0) software was utilised to analyse all ncRNAs and mRNAs based on a negative binomial distribution model (Love et al. 2014). Heat map and bar chart were constructed using TBtools (version 2.112) and GraphPadPrism9, respectively, based on the expression profiles of significantly DEMs, DEGs, and DELs. Additionally, an upset diagram was generated using the OmicShare online platform (http://www.omicshare.com/tools).

### 4.5 Functional Annotation of Target Genes

The identification of *cis*‐target genes was performed using LncTar (http://www.cuilab.cn/lnctar). The prediction of *cis*‐regulated target genes for differentially expressed lncRNAs was conducted, while miRNAs target genes were predicted using the psRobot software (version 1.2) (Wu et al. 2012). Gene abbreviations were obtained from the Ensemble database (https://plants.ensembl.org/index.html). Background annotation information for Gene Ontology (GO) or Kyoto Encyclopedia of Genes and Genomes (KEGG) pathways in grape was obtained from the Ensemble website ( *Vitis vinifera* PN40024.v4.53). Functional enrichment analysis of GO and KEGG pathways for all *cis*‐target genes of DELs and target genes of DEMs was performed using the OmicShare platform. GO enrichment maps of multiple groups were drawn using the ggplot2 package in R (verision4.3.1). KEGG pathway analysis results were visualised using the online platform (https://www.bioinformatics.com.cn/).

### 4.6 CeRNA Network Analysis

DELs were predicted as DEMs targets using psRobot. All DELs, DEMs and DEGs were used for ceRNA prediction. Based on the predicted results of lncRNAs‐miRNAs‐mRNAs, the ceRNA networks diagram was drawn by Cytoscape (version 3.7.1).

### 4.7 m^6^A Methylation Sequencing and Data Analysis

The mRNA enriched by Dynabeads Oligo (dT, Thermo Fisher, USA) was fragmented into short segments using Magnesium RNA Fragmentation Module (NEB, 6150, USA) and subjected to co‐immunoprecipitated (IP) with an m^6^A‐specific antibody (Synaptic Systems). Subsequent steps and methodologies were performed as previously described. The resulting library products were sequenced on the Illumina NovaseqTM 6000 platform using the Illumina PE150 strategy. Raw reads containing adapters, polyA/polyG or low‐quality bases were filtered out using Fastp software (Chen et al. 2018). Clean reads were then mapped to the grapevine genome ( *Vitis vinifera* PN40024.v4.53) using HISAT2 (version 2.2.1) (Kim et al. 2019). Exomepeak was employed to identify intergroup peaks and analyse differentially methylated m^6^A peak (DMPs) between groups (Meng et al. 2014). The identified m^6^A peaks were annotated using ANNOVAR (https://annovar.openbioinformatics.org). HOMER (version 4.10) was used to predict common motifs region (Heinz et al. 2010). Common motif regions were predicted using DESeq2, with significance threshold of *p*‐value < 0.05 and |log2FC| > 1. Genes associated DMPs (DEMGs) were further subjected to GO and KEGG enrichment analysis (http://www.geneontology.org/). Visualisation of m^6^A peaks was conducted using Integrative Genomics Viewer (IGV, version 2.12.3) (Robinson et al. 2017).

### 4.8 Real‐Time Fluorescence Quantitative Analysis

Total RNA was extracted from *V. quinquangularis* and *V. vinifera* grape leaves using the Plant Total RNA Kit (Omega‐Bio‐Tek, China). Subsequently, the RNA was reverse‐transcribed into cDNA for real‐time fluorescence quantitative (RT‐qPCR) analysis. For miRNA analysis, cDNA synthesis was performed using the miRcute Plus miRNA First Strand cDNA Synthesis Kit (Tiangen, China), followed by quantitative PCR using the miRcute miRNA qPCR detection kit (Tiangen, China). The expression of lncRNAs was quantified using ChamQ SYBR Green Master Mix (Vazyme, Nanjing, China). All primers used in this study are listed in Table S21 and were synthesised by Qingke Jersey Biotechnology Co. Ltd. (Xi'an, China). MiR168 served as the internal control for miRNA RT‐qPCR analysis, and *EF1‐α* was used as the reference gene for mRNA and lncRNA quantification, as described in a previous study (Luo et al. 2024). Each sample has three independent biological replicates, each with three technical replicates.

### 4.9 Transient Expression in *V. quinquangularis* Leaves

We performed *Agrobacterium*‐mediated transient expression in *V. quinquangularis* leaves following the methodology described by Luo et al. (2025). Fully expanded leaves with similar growth status were collected from *V. quinquangularis.* *Agrobacterium tumefaciens* cells harbouring pCAMBIA2300: GFP or pCAMBIA2300: LRR‐RLK‐GFP constructs were cultured in Luria‐Bertani broth until the Optical Density at 600 nm (OD_600_) reached 0.6. Young and fully expanded leaves were placed in a culture dish containing 30 mL of the bacterial suspension with the abaxial side facing downward. The leaves were subjected to vacuum infiltration for 30 min, followed by incubation for 48 h. After incubation, the infiltrated leaves were collected for *Coniella diplodiella* infection and further RT‐qPCR validation. The *C. diplodiella* infection assays were performed according to the established protocol by Tan et al. (2024). Each sample has three independent biological replicates, each with three technical replicates.

### 4.10 Phenotype and RT‐qPCR Analysis of *V. quinquangularis* Leaves After Over‐Expression of lncRNAs via *Agrobacterium*‐Mediated Transient Transformation

The transcript sequence of lncRNAs was obtained from the SY grape DNA library by using Phanta Max Super‐Fidelity DNA Polymerase (Vazyme) with the primers listed in supplementary Table 21. The DNA library was obtained by Universal Genomic DNA Kit (CWBIO). Same as the above described, the PCR products of lncRNAs were introduced into pCAMBIA‐1301 (Miaolingbio, Wuhan, China) to obtain OE‐lncRNAs, and these vectors were introduced into *V. quinquangularis* leaves using *Agrobacterium*‐mediated transient transformation for 48 h. After incubation, the infiltrated leaves were collected for *C. diplodiella* infection and further RT‐qPCR validation. All RT‐qPCR analyses in our study were performed with three independent biological replicates, each with three technical replicates.

## Author Contributions

D.D. designed and conceptualised this work. R.D. verified and analysed the data, as well as drafted the manuscript. R.Z. conducted several experiments. Y.F., W.L., and Q.X. contributed to various analyses and assisted in the collection of grapevine leaves. D.D. subsequently revised this manuscript. Finally, all authors gave final approval for the submission of the paper.

## Conflicts of Interest

The authors declare no conflicts of interest.

## Supporting information


**Figure S1:** Differentially expressed miRNAs under flg22 treatment identified in different samples.
**Figure S2:** The TPM value of differentially expressed miRNAs.
**Figure S3:** Functional analysis of differentially expressed miRNAs in different groups. (a–d) KEGG analysis of miRNA target genes in different groups. (e) The heat map for miRNAs target genes.
**Figure S4:** Number of DELs in different groups.
**Figure S5:** Characteristics of differentially expressed lncRNAs in grapevine. (a) The expression level of differentially expressed lncRNAs. (b) The expression pattern of differentially expressed lncRNAs verified by RT‐qPCR. (c) Heat map of differentially expressed lncRNAs with higher expression in SY_6 than CS_6 and their *cis*‐target genes. Heatmap was plotted with FPKM values and averaged over three biological replicates for each sample. *WRKY31* (Vitvi10g00063), WRKY transcription factor 31; *BRI1* (Vitvi12g01199, Vitvi12g01277, Vitvi12g01323) Brassinosteroid insensitive 1‐associated receptor kinase 1; *CBL1* (Vitvi13g04597), calcineurin B‐like protein 1; *COL7* (Vitvi10g00219), zinc finger protein CONSTANS‐LIKE 9; *CRLK2* (Vitvi14g01319), calcium/calmodulin‐regulated receptor‐like kinase 2; *EMS1* (Vitvi16g00979), leucine‐rich repeat receptor protein kinase EMS1; *GST* (Vitvi05g04186), glutathione S‐transferase; *LECRK59* (Vitvi13g04016, Vitvi13g00095), L‐type lectin‐domain containing receptor kinase V.9‐like; *LRR‐RLK* (Vitvi05g00459, Vitvi09g01335, Vitvi10g00652, Vitvi12g04366), LRR receptor‐like serine/threonine protein kinase; *MAPKKK* (Vitvi04g01691), mitogen‐activated protein kinase; MYB4 (Vitvi05g01732), myb‐related protein Myb4; *RALF33* (Vitvi14g00168), protein RALF‐like 33; *RBK2* (Vitvi05g00384), receptor‐like cytosolic serine/threonine‐protein kinase RBK2; *RBL2* (Vitvi02g00382), RHOMBOID‐like protein 2; *RF9* (Vitvi15g00331)/*RGA3* (Vitvi19g01617)/*RGA4* (Vitvi19g04569)/*RPP13L4* (Vitvi19g04136), disease resistance protein RF9/RGA3/4/RPP13L4; *RIN4* (Vitvi05g01136), RPM1‐interacting protein 4. *RLK* (Vitvi10g04213), receptor‐like protein kinase; *SBT* (Vitvi07g01734), subtilisin‐like protease SBT; *SD1‐8* (Vitvi19g01931, Vitvi19g04149, Vitvi19g04150, Vitvi19g04155), receptor‐like serine/threonine‐protein kinase SD1‐8; *STS* (Vitvi16g01485, Vitvi16g04347, Vitvi16g04353, Vitvi16g04348), stilbene synthase; *TLP* (Vitvi02g04092), thaumatin‐like protein; ZNF8 (Vitvi03g01288), zinc transporter 8. The arrow indicates the targeting relation.
**Figure S6:** Functional analysis of *cis*‐target genes of differentially expressed lncRNAs. (a–d) The KEGG pathways of *cis*‐target genes of DELs in different groups. (e) The heat map for target genes of lncRNAs.
**Figure S7:** The quality of the RNA‐seq data. (a and b) The three independent biological replicates for each sample and correlation analysis between the different samples in RNA‐seq.
**Figure S8:** Distribution of clean reads in all samples.
**Figure S9:** Distribution of all samples peaks in transcription start and end sites.
**Figure S10:** GO and KEGG enrichment analysis of hypermethylated and hypomethylated modification genes.
**Figure S11:** Combined analysis of m^6^A‐seq and RNA‐seq. (a) Volcano plot of the differential genes in RNA‐seq. (b) Venn plot of the differentially methylated modified genes and the differentially expressed genes in SY_6 vs. SY_0 h. (c) GO term assignments of all differential m^6^A‐methylated DEGs. (d) KEGG enrichment term of all differential m^6^A‐methylated DEGs.

**Table S1:** MiRNA‐seq summary information of all sequence samples.
**Table S2:** LncRNA‐seq summary information of all sequence samples.
**Table S3:** NcRNA summary information of two grape varieties.
**Table S4:** sRNA classification statistics data.
**Table S5:** Significant enriched GO term of differently expressed miRNAs target genes.
**Table S6:** Functional annotation of DEMs' target genes in four clusters.
**Table S7:** KEGG term of DEMs' target genes.
**Table S8:** Summary of grape lncRNA sequencing data.
**Table S9:** Summary of CPC, CNCI, CPAT and Pfam analyses.
**Table S10:** Comparative analysis of lncRNA and mRNA transcript length and exon number.
**Table S11:** *Cis*‐target genes prediction of differentially expressed lncRNAs in different samples.
**Table S12:** Significant enriched GO term of DELs *cis*‐target genes.
**Table S13:** Functional annotation of resistance‐related *cis*‐target genes in four clusters.
**Table S14:** Construction of ce‐RNA network.
**Table S15:** Summary of the sequencing results obtained from m^6^A‐seq conducted in this study.
**Table S16:** Summary of mapped reads in m^6^A‐seq obtained from this study.
**Table S17:** Numbers of the m^6^A‐modified transcripts and peak annotation in grape samples.
**Table S18:** Data description of differentially methylated peaks in this study.
**Table S19:** GO enrichment of methylated modification genes.
**Table S20:** GO analysis of DEMGs.
**Table S21:** The primer sequences for RT‐qPCR and PCR.

## Data Availability

The raw data of MeRIP‐seq, miRNAs and lncRNAs had been uploaded to the NCBI SRA public database under the numbers PRJNA1210810, PRJNA1144342 and PRJNA1144261, respectively (https://submit.ncbi.nlm.nih.gov/subs/sra/). All pertinent data can be found within this article.
