# Supplementary material for: Analysis of Non‐Coding RNAs and N6‐Methyladenosine‐Modified Genes in Response to flg22 in Grape Immunity
Source: Plant Biotechnol J. 2025 Aug 17;23(12):5435–55. doi: 10.1111/pbi.70303 (PMC12665093; doi:10.1111/pbi.70303)
Supplement: Supplementary file 1 — Figure S1: Differentially expressed miRNAs under flg22 treatment identified in different samples. Figure S2: The TPM value of differentially expressed miRNAs. Figure S3: Functional analysis of differentially expressed miRNAs in different groups. (a–d) KEGG analysis of miRNA target genes in different groups. (e) The heat map for miRNAs target genes. Figure S4: Number of DELs in different groups. Figure S5: Characteristics of differentially expressed lncRNAs in grapevine. (a) The expression level of differentially expressed lncRNAs. (b) The expression pattern of differentially expressed lncRNAs verified by RT‐qPCR. (c) Heat map of differentially expressed lncRNAs with higher expression in SY_6 than CS_6 and their cis‐target genes. Heatmap was plotted with FPKM values and averaged over three biological replicates for each sample. WRKY31 (Vitvi10g00063), WRKY transcription factor 31; BRI1 (Vitvi12g01199, Vitvi12g01277, Vitvi12g01323) Brassinosteroid insensitive 1‐associated receptor kinase 1; CBL1 (Vitvi13g04597), calcineurin B‐like protein 1; COL7 (Vitvi10g00219), zinc finger protein CONSTANS‐LIKE 9; CRLK2 (Vitvi14g01319), calcium/calmodulin‐regulated receptor‐like kinase 2; EMS1 (Vitvi16g00979), leucine‐rich repeat receptor protein kinase EMS1; GST (Vitvi05g04186), glutathione S‐transferase; LECRK59 (Vitvi13g04016, Vitvi13g00095), L‐type lectin‐domain containing receptor kinase V.9‐like; LRR‐RLK (Vitvi05g00459, Vitvi09g01335, Vitvi10g00652, Vitvi12g04366), LRR receptor‐like serine/threonine protein kinase; MAPKKK (Vitvi04g01691), mitogen‐activated protein kinase; MYB4 (Vitvi05g01732), myb‐related protein Myb4; RALF33 (Vitvi14g00168), protein RALF‐like 33; RBK2 (Vitvi05g00384), receptor‐like cytosolic serine/threonine‐protein kinase RBK2; RBL2 (Vitvi02g00382), RHOMBOID‐like protein 2; RF9 (Vitvi15g00331)/RGA3 (Vitvi19g01617)/RGA4 (Vitvi19g04569)/RPP13L4 (Vitvi19g04136), disease resistance protein RF9/RGA3/4/RPP13L4; RIN4 (Vitvi05g01136), RPM1‐interacting protein 4. RLK [file PBI-23-5435-s002.docx]

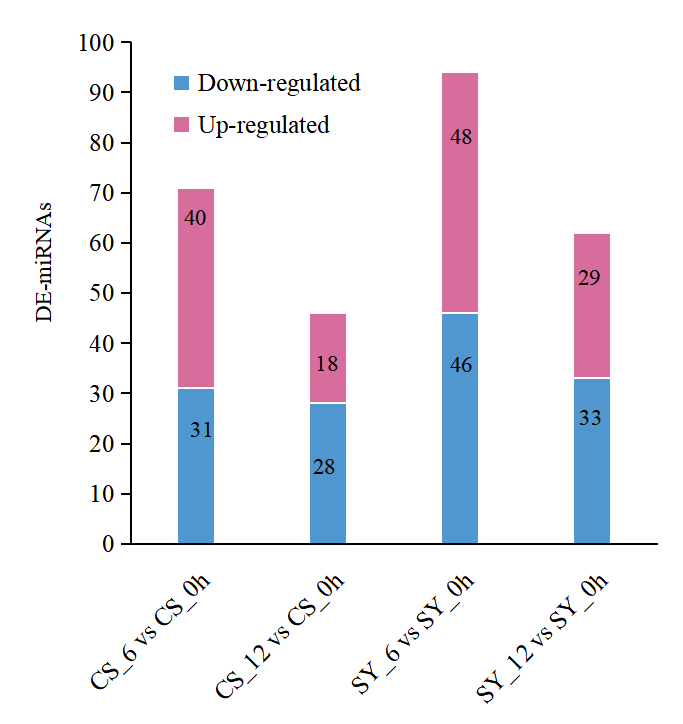


Figure S1. Differentially expressed miRNAs under flg22 treatment identified in different samples.


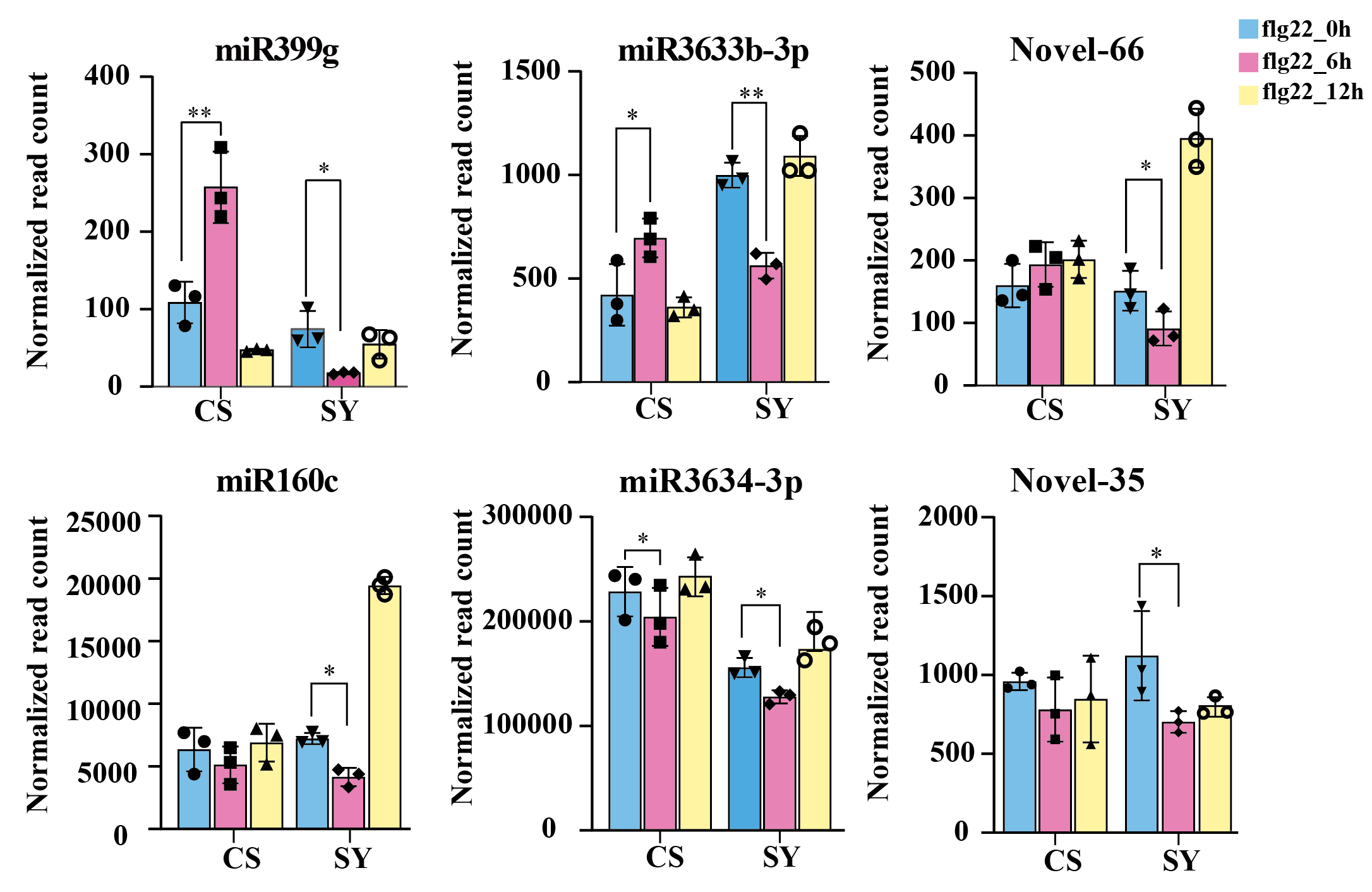


Figure S2. The TPM value of differentially expressed miRNAs.


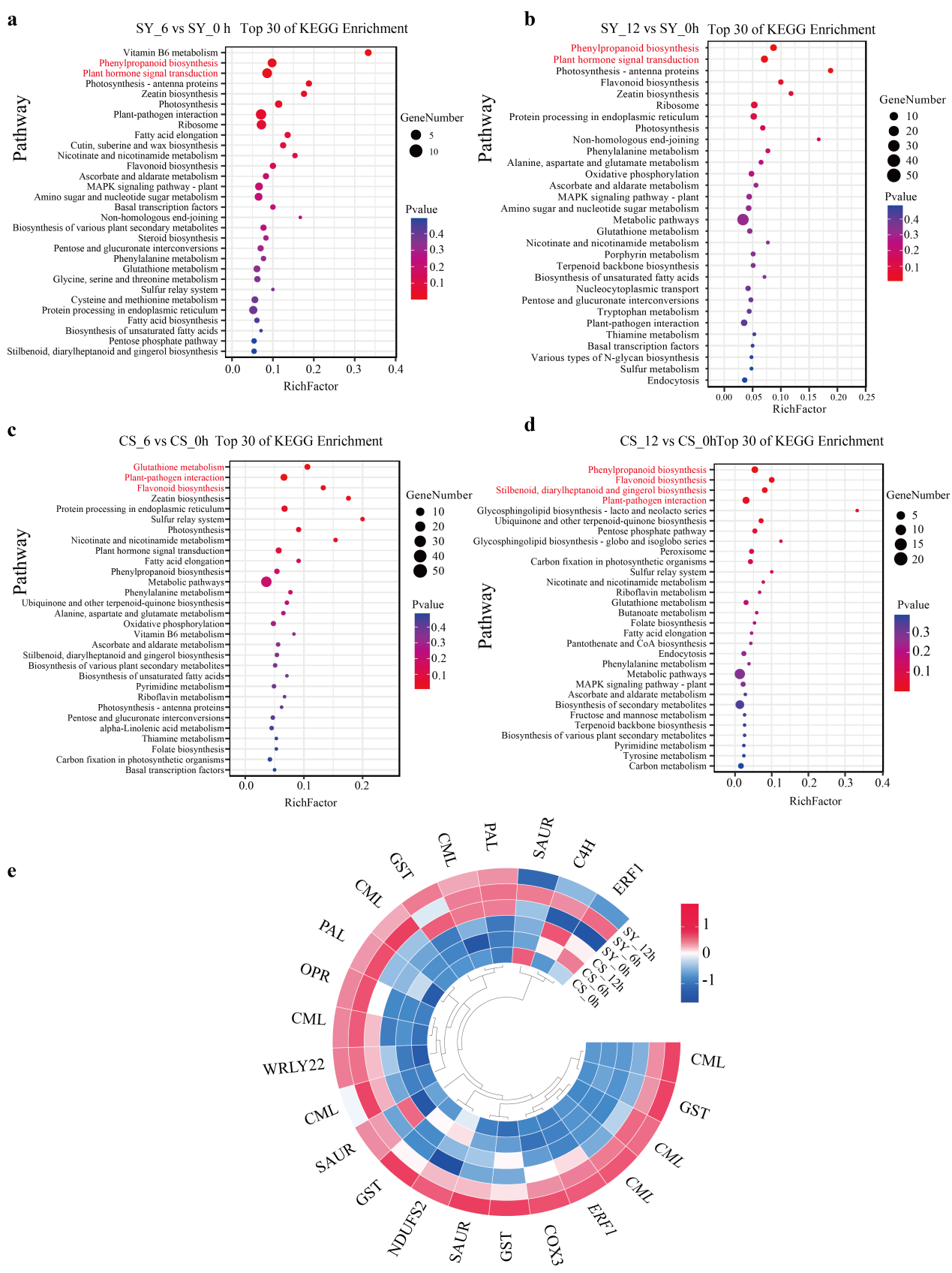


Figure S3. Functional analysis of differentially expressed miRNAs in different groups. **a-d**) KEGG analysis of miRNA target genes in different groups. **e**) The heat map for miRNAs target genes.


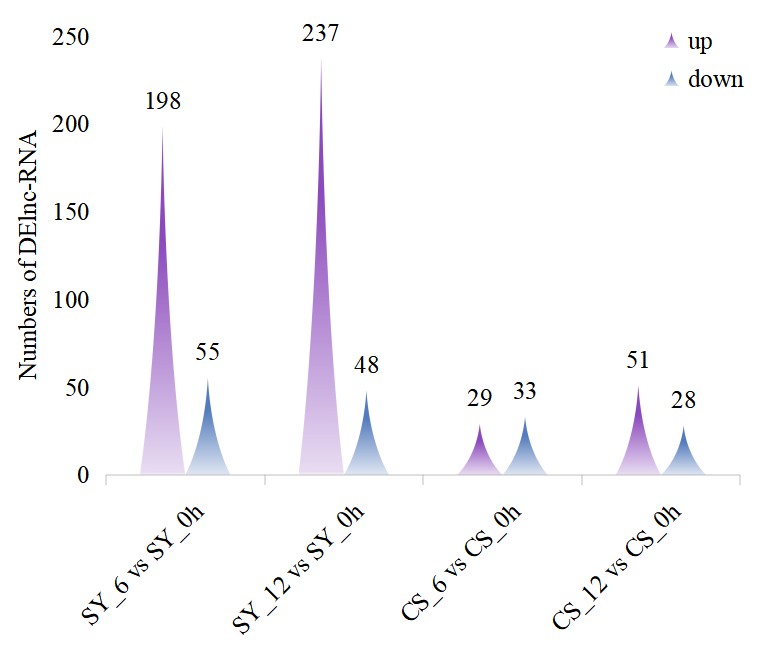


Figure S4. Number of DELs in different groups.


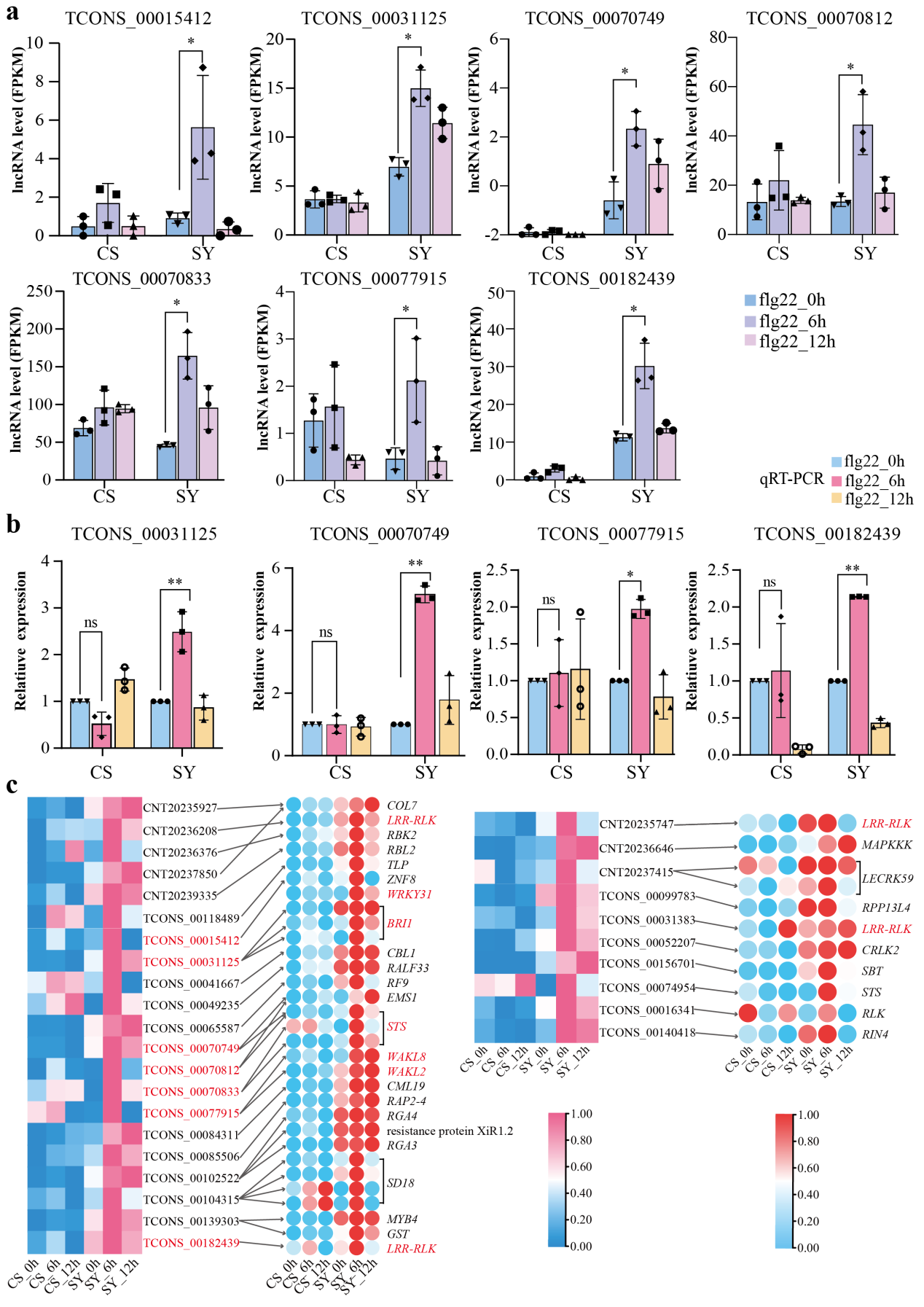


Figure S5. Characteristics of differentially expressed lncRNAs in grapevine. **a)** The expression level of differentially expressed lncRNAs. **b)** The expression pattern of differentially expressed lncRNAs verified by RT-qPCR. **c)** Heat map of differentially expressed lncRNAs with higher expression in SY_6 than CS_6 and their *cis*-target genes. Heatmap was plotted with FPKM values and averaged over three biological replicates for each sample. *WRKY31* (Vitvi10g00063), WRKY transcription factor 31; *BRI1* (Vitvi12g01199, Vitvi12g01277, Vitvi12g01323) Brassinosteroid insensitive 1‐associated receptor kinase 1; *CBL1* (Vitvi13g04597), calcineurin B‐like protein 1; *COL7* (Vitvi10g00219), zinc finger protein CONSTANS‐LIKE 9; *CRLK2* (Vitvi14g01319), calcium/calmodulin‐regulated receptor‐like kinase 2; *EMS1* (Vitvi16g00979), leucine‐rich repeat receptor protein kinase EMS1; *GST* (Vitvi05g04186), glutathione S‐transferase; *LECRK59* (Vitvi13g04016, Vitvi13g00095), L‐type lectin‐domain containing receptor kinase V.9‐like; *LRR‐RLK* (Vitvi05g00459, Vitvi09g01335, Vitvi10g00652, Vitvi12g04366), LRR receptor‐like serine/threonine protein kinase; *MAPKKK* (Vitvi04g01691), mitogen‐activated protein kinase; *MYB4* (Vitvi05g01732), myb‐related protein Myb4; *RALF33* (Vitvi14g00168), protein RALF‐like 33; *RBK2* (Vitvi05g00384), receptor‐like cytosolic serine/threonine‐protein kinase RBK2; *RBL2* (Vitvi02g00382), RHOMBOID‐like protein 2; *RF9* (Vitvi15g00331)/ *RGA3* (Vitvi19g01617)/ *RGA4* (Vitvi19g04569)/ *RPP13L4* (Vitvi19g04136), disease resistance protein RF9/RGA3/4/RPP13L4; *RIN4* (Vitvi05g01136), RPM1‐interacting protein 4. *RLK* (Vitvi10g04213), receptor‐like protein kinase; *SBT* (Vitvi07g01734), subtilisin‐like protease SBT; *SD1-8* (Vitvi19g01931, Vitvi19g04149, Vitvi19g04150, Vitvi19g04155), receptor‐like serine/threonine‐protein kinase SD1‐8; *STS* (Vitvi16g01485, Vitvi16g04347, Vitvi16g04353, Vitvi16g04348), stilbene synthase; *TLP* (Vitvi02g04092), thaumatin‐like protein; *ZNF8* (Vitvi03g01288), zinc transporter 8. The arrow indicates the targeting relation.


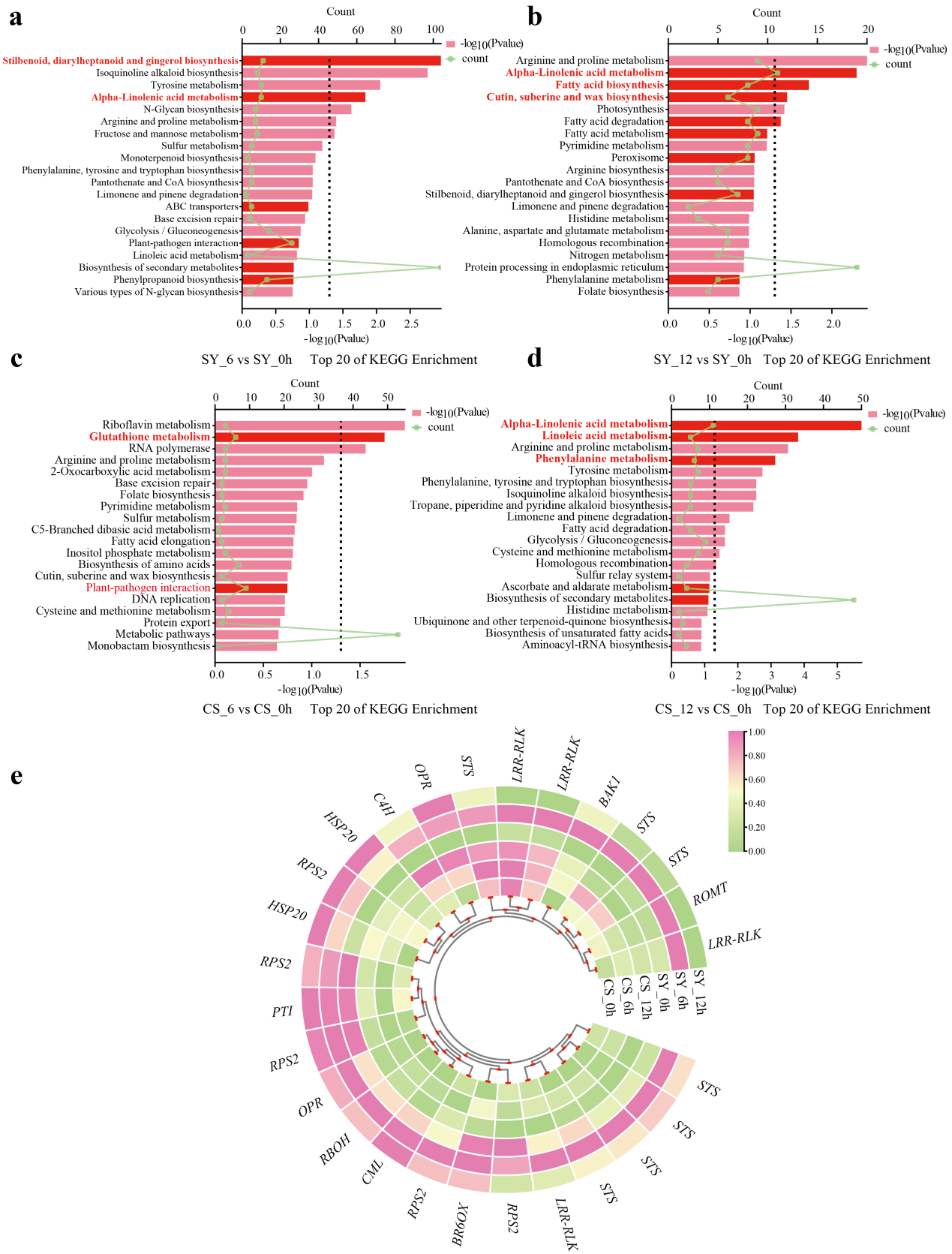


Figure S6. Functional analysis of *cis*-target genes of differentially expressed lncRNAs. **a-d)** The KEGG pathways of *cis*-target genes of DELs in different groups. **e)** The heat map for target genes of lncRNAs.


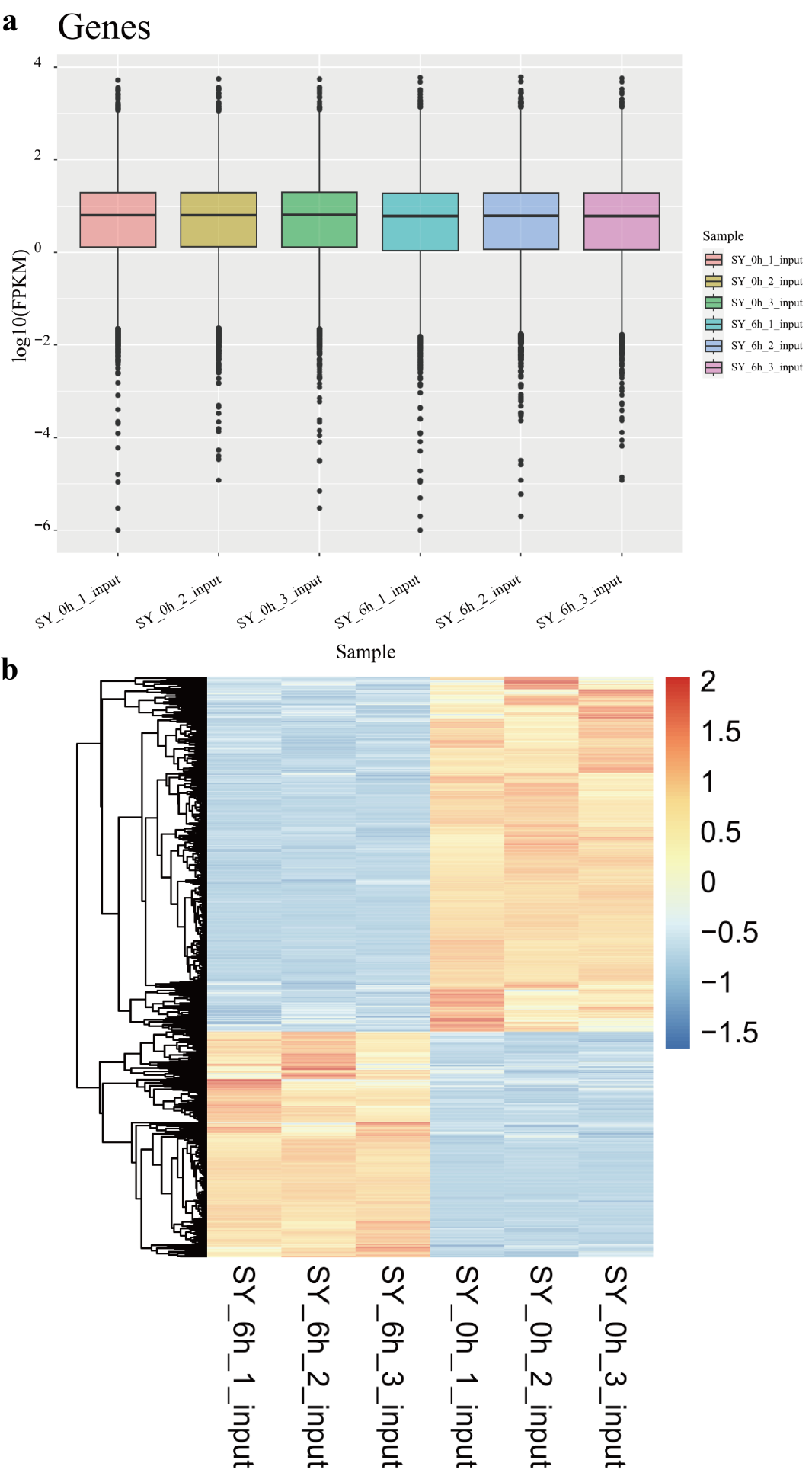


Figure S7. The quality of the RNA-seq data. **a and b)** The three independent biological replicates for each sample and correlation analysis between the different samples in RNA-seq.


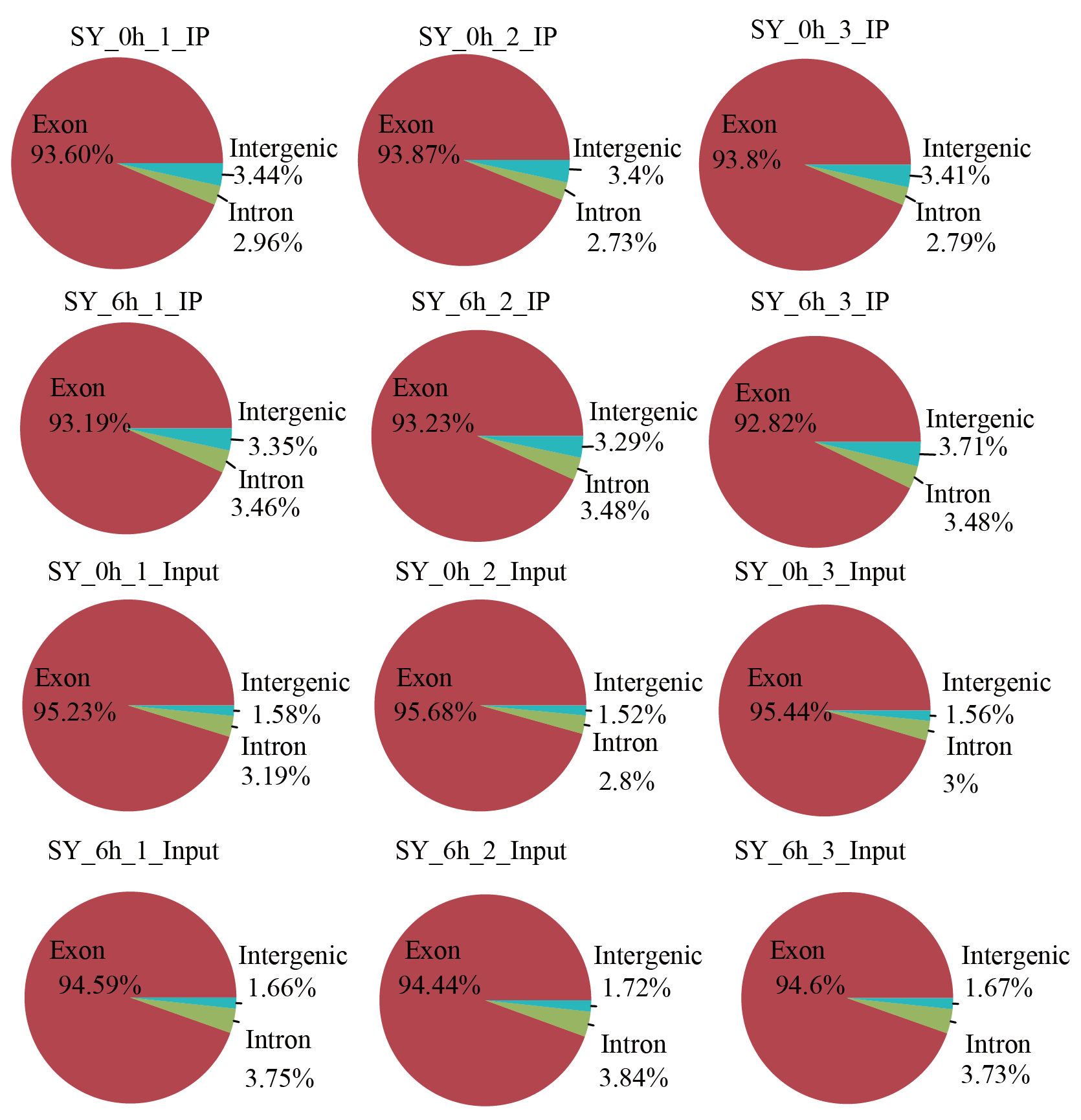


Figure S8. Distribution of clean reads in all samples.


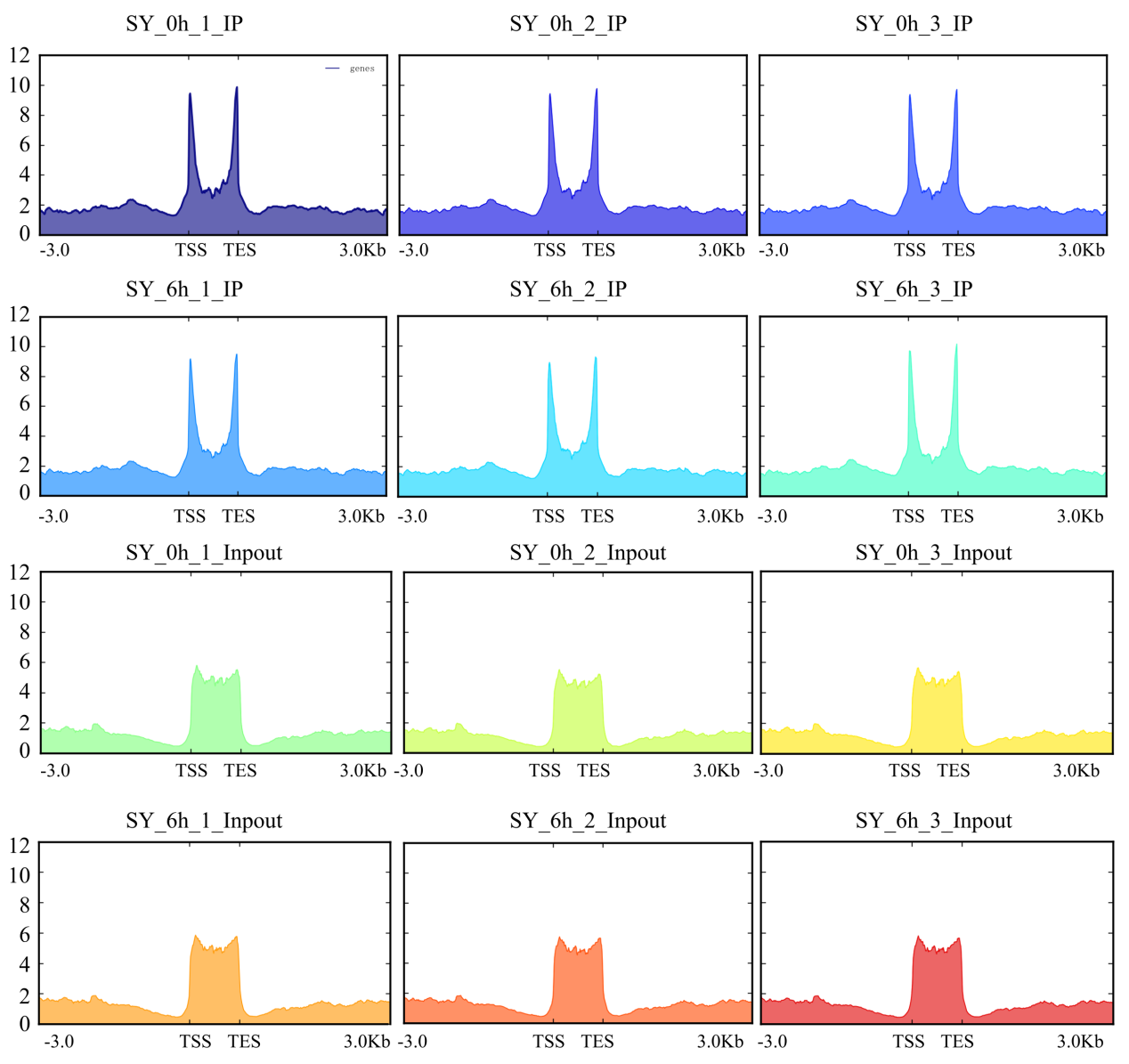


Figure S9. Distribution of all samples peaks in transcription start and end sites.


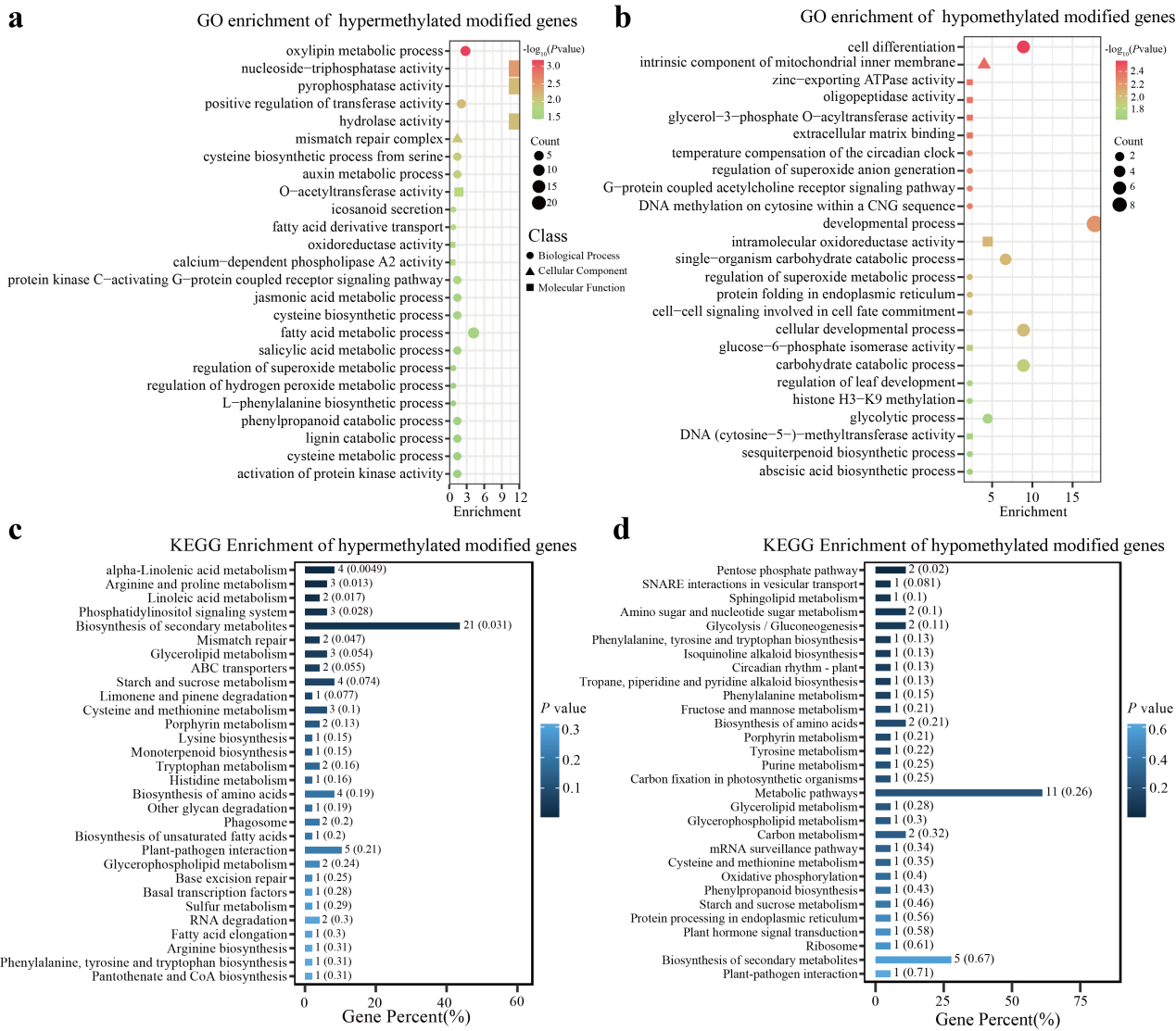


Figure S10. GO and KEGG enrichment analysis of hypermethylated and hypomethylated modification genes.


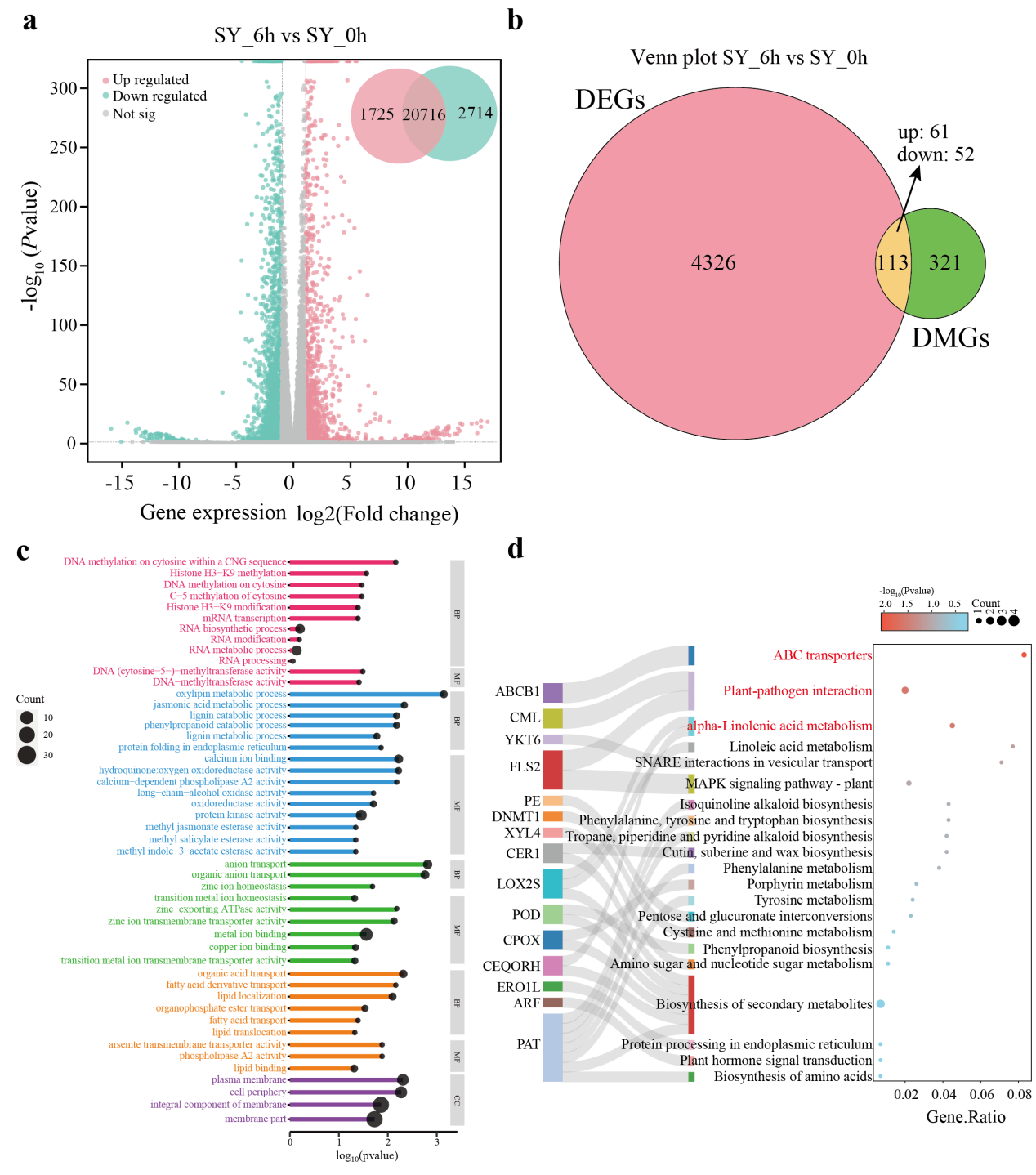


Figure S11. Combined analysis of m^6^A-seq and RNA-seq. **a)** Volcano plot of the differential genes in RNA-seq. **b)** Venn plot of the differentially methylated modified genes and the differentially expressed genes in SY_6 vs. SY_0 h. **c)** GO term assignments of all differential m^6^A-methylated DEGs. **d)** KEGG enrichment term of all differential m^6^A-methylated DEGs.
